# Anti-SARS-CoV-2 equine F (Ab′)_2_ immunoglobulin as a possible therapy for COVID-19

**DOI:** 10.1038/s41598-022-07793-1

**Published:** 2022-03-10

**Authors:** Viviane Fongaro Botosso, Soraia Attie Calil Jorge, Renato Mancini Astray, Ana Marcia de Sá Guimarães, Monica Beatriz Mathor, Patrícia dos Santos de Carneiro, Edison Luiz Durigon, Dimas Covas, Danielle Bruna Leal de Oliveira, Ricardo das Neves Oliveira, Durvanei Augusto Maria, Silas Fernandes Eto, Neuza Maria Frazatti Gallina, Giselle Pidde, Carla Cristina Squaiella-Baptistão, Dilza Trevisan Silva, Isadora Maria Villas-Boas, Dayanne Carla Fernandes, Aline Vivian Vatti Auada, Alexandre Campos Banari, Antônio Francisco de Souza Filho, Camila Bianconi, Carla Lilian de Agostini Utescher, Denise Cristina André Oliveira, Douglas Oscar Ceolin Mariano, Flávia Ferreira Barbosa, Giuliana Rondon, Josana Kapronezai, Juliana Galvão da Silva, Mauricio Barbugiani Goldfeder, Priscila Comone, Regis Edgar Castilho Junior, Taiana Tainá Silva Pereira, Fan Hui Wen, Denise V. Tambourgi, Ana Marisa Chudzinski-Tavassi

**Affiliations:** 1 Virology Laboratory, Butantan Institute, São Paulo, Brazil; 2 Viral Biotechnology Laboratory, Butantan Institute, São Paulo, Brazil; 3 Multipurpose Laboratory, Butantan Institute, São Paulo, Brazil; 4 Microbiology Department, Biomedical Science Institute, University of São Paulo, São Paulo, Brazil; 5 Institute for Energy and Nuclear Research, São Paulo, Brazil; 6 Quality Control, Butantan Institute, São Paulo, Brazil; 7 Direction, Butantan Institute, São Paulo, Brazil; 8 Bioindustrial Center, Butantan Institute, São Paulo, Brazil; 9 Development and Innovation Laboratory, Butantan Institute, São Paulo, Brazil; 10 Viral Vaccines Pilot Laboratory, Butantan Institute, São Paulo, Brazil; 11 Immunochemistry Laboratory, Butantan Institute, São Paulo, Brazil; 12 Center of Excellence in New Target Discovery (CENTD), Special Laboratory, São Paulo, Brazil

**Keywords:** Diseases, Viral infection, Applied immunology

## Abstract

The new outbreak of coronavirus disease 2019 (COVID-19) has infected and caused the death of millions of people worldwide. Intensive efforts are underway around the world to establish effective treatments. Immunoglobulin from immunized animals or plasma from convalescent patients might constitute a specific treatment to guarantee the neutralization of the virus in the early stages of infection, especially in patients with risk factors and a high probability of progressing to severe disease. Worldwide, a few clinical trials using anti-SARS-CoV-2 immunoglobulins from horses immunized with the entire spike protein or fragments of it in the treatment of patients with COVID-19 are underway. Here, we describe the development of an anti-SARS-CoV-2 equine F(ab′)_2_ immunoglobulin using a newly developed SARS-CoV-2 viral antigen that was purified and inactivated by radiation. Cell-based and preclinical assays showed that the F(ab′)_2_ immunoglobulin successfully neutralizes the virus, is safe in animal models, and reduces the severity of the disease in a hamster model of SARS-CoV-2 infection and disease.

## Introduction

In late 2019, China reported the first case of COVID-19 (coronavirus disease 2019), a severe acute respiratory syndrome caused by SARS-CoV-2 (severe acute respiratory syndrome coronavirus-2). The new virus spread rapidly throughout the world, reaching 275 million cases and 5.3 million deaths as of December 2021^1^. Although vaccines became available in less than a year, approaches for COVID-19 treatment remain scarce. Many studies have sought to develop different therapeutic interventions, ranging from the use of antiviral and antiparasitic drugs to immunotherapy with interleukins (e.g., interferon-alpha), natural killer cells or mesenchymal stem cells, as well as the transfusion of convalescent patient plasma and specific monoclonal antibodies^2^.

The use of convalescent plasma was one of the most widely studied therapies because of the history of success for the treatment of other infectious diseases and, in addition, it was an easier source of specific antibodies. However, the results from randomized controlled clinical trials involving convalescent plasma transfusion in patients with COVID-19 are mixed^3,4^. Furthermore, although promising, transfusion of convalescent plasma has important limitations.

First, the ability to obtain plasma depends on the voluntary donation of convalescent patients. In addition, substantial heterogeneity in the potency of the antibodies generated among individuals has been observed that could lead to less effective treatment.

These disadvantages can be overcome using heterologous antibodies obtained from the plasma of horses immunized with SARS-CoV-2. Among other aspects, the use of equine immunoglobulins has the advantages of obtaining antibodies for long periods, as horses receive periodic immunization boosters and produce large amounts of plasma, and researchers have greater control in obtaining homogeneous final preparations. The final product consists of highly purified antibodies, not raw plasma, providing greater safety and efficacy. In addition, enzymatic digestion of the immunoglobulin molecule to remove the Fc fraction can be performed without loss of neutralizing capacity conferred by the Fab portion of the immunoglobulin.

Preliminary results showed that equine antibody therapy was well tolerated, as adverse reactions were mild and moderate, and hospitalized patients presented a clinical improvement of SARS-CoV-2-induced pneumonia, especially those with severe disease^5^. In parallel, two different IgG formulations have been prepared from the plasma of horses immunized with S1 protein or a mixture of SARS-CoV-2 recombinant proteins (S1, N, and SEM), and both exhibited high in vitro neutralizing capacity^6^. A clinical trial is underway to compare the safety and efficacy of the anti-S1 equine antibody formulation to treat moderate and severe COVID-19 cases^7^.

All these studies used the entire spike protein or fragments of it, combined with or without other recombinant proteins of SARS-CoV-2, to immunize the horses, as the spike protein is responsible for the attachment of the virus to the human ACE2 (angiotensin converting enzyme 2) receptor^8^. None of these formulations have used the entire virus, which would theoretically produce additional antibodies that may halt other viral processes and help fight infection. Therefore, considering the urgent need for a specific treatment for COVID-19, the aim of this study was to develop an anti-SARS-CoV-2 equine immunoglobulin F(ab′)_2_ for the treatment of COVID-19 using a newly developed SARS-CoV-2 viral antigen that was purified and inactivated by radiation. We describe that this novel serum, which was developed as an end product for clinical use, successfully neutralizes the virus, is safe in animal models, and is able to reduce the severity of the disease in a hamster model of SARS-CoV-2 infection and disease.

## Materials and methods

### Production of purified and inactivated SARS-CoV-2

*SARS-CoV-2 Bank:* SARS-CoV-2/SP02/2020HIAE (GenBank MT126808.1. B.1.1.28) is a clinical isolate from one of the first patients diagnosed with COVID-19 in Brazil, admitted to the Hospital Israelista Albert Einstein (São Paulo, Brazil). The clinical material was tested for SARS-CoV-2^9^ and for 15 viral agents using real-time PCR (influenza A and B, endemic coronavirus—CoV-NL63, -229E, -HKU1 and -OC43, enterovirus, parainfluenza virus 1, 2, 3 and 4; human metapneumovirus, rhinovirus, respiratory syncytial virus and adenovirus)^10^. No viral agent, except for SARS-CoV-2, was detected in the sample. The virus was isolated in VERO E6 cells, and the characterization of the strain was described by Araujo et al.^10^. A nucleotide identity comparison between the genomes of the used strain (GenBank MT126808.1. B.1.1.28) and the original Wuhan-Hu-1 genome (NC_045512.2) was also performed. Their whole genome identity is 99.99%, with only four different nucleotides and no changes in the amino acid sequence of the spike protein. Working virus seed stock (WVSS) was prepared by infecting VERO CCL-81.4 cells (ECAAC General Collection 88020401) adapted to VP serum-free medium (Thermo Fisher, MA, USA) at Butantan Institute with an MOI (multiplicity of infection) of 0.1 for 48 h at 37 °C in a 5% CO_2_ atmosphere. The produced virus was filter-sterilized (0.22 µm), formulated with SPG (7.462 g/L sucrose, 0.0517 g/L KH_2_PO_4_, 0.1643 g/L K_2_HPO_4_·3 H_2_O and 0.0907 g/L potassium glutamate) and frozen at − 80 °C. The final WVSS titre was 9.79E + 06 TCID_50_/mL. All manipulations of the active virus were performed in a BSL3 laboratory.

#### Virus titration

Determination of the 50% Tissue Culture Infective Dose (TCID_50_): Virus samples were taken from culture or purification steps and immediately frozen at − 80 °C before analysis. After thawing, samples were serially diluted ten-fold (10^–1^–10^–12^) with Dulbecco’s modified Eagle’s medium (DMEM) containing 2.5% foetal bovine serum (FBS) and inoculated in six replicates in 96-well plates in which VERO ATCC CCL-81.4 cells were seeded (2 × 10E + 04 cells/well). After an incubation at 37 °C with 5% CO_2_ for 72 h, the plates were microscopically inspected for cytopathic effects (CPE) caused by SARS-CoV-2. The monolayers were fixed and stained with a Naphthol Blue Black (Sigma-Aldrich) solution (0.1% naphthol blue w/w with 5.4% acetic acid and 0.7% sodium acetate) and analysed to confirm the results. The viral titre was calculated using the Spearman & Kärber algorithm^11^ and reported in TCID_50_/mL.

#### Antigen production and inactivation

For antigen production, Vero ATCC CCL-81.4 cells were seeded in T 225 cm^2^ flasks (107 cells/flask) and grown for 48 h before infection. SARS-CoV-2/SP02/2020HIAE was inoculated at an MOI of 0.1 in 5 mL of culture medium and allowed to adsorb for one hour at 37 °C. Serum-free medium was added to cultures to a volume of 100 mL. The cultures were incubated at 37 °C with 5% CO_2_ for 48 h. Before harvest, flasks were microscopically inspected for cytopathic effects and the absence of contamination, and then the culture medium was transferred to a new flask. Three batches of active SARS-CoV-2 were clarified through 0.22 µm filters (Opticap XL2 Durapore, Merck Millipore) and pooled (6 L) for the following purification process. Clarified material was concentrated by tangential flow filtration (TFF) using a Mini Pellicon system with an Ultracell 300 kDa membrane (Merck Millipore). The concentrated material (2 L) was subjected to two rounds of ultracentrifugation (XPN 90, Beckman Coulter) in sucrose gradients (65–34%) using a zonal Ti15 rotor for 3 h at 4 °C and 25 krpm. Fractions containing the purified virus were pooled after each ultracentrifugation procedure. The final purified material was diluted with 1× PBS to a sucrose concentration of 20%, filter-sterilized (0.22 µm) and frozen at − 80 °C. Frozen aliquots of purified SARS-CoV-2 were subjected to virus inactivation using gamma irradiation at Instituto de Pesquisas Nucleares (IPEN, São Paulo, Brazil) using GammaCell Irradiation unit, (Nordion, Canada). The radiation dose for virus inactivation was previously chosen through a D10 experiment where several doses were used to reduce the virus titer proportionally. The gamma radiation dose necessary to inactivate SARS-CoV-2 (Wuhan strain) was determined as 15 kGy already considering the sterilization assurance level required for animal or pharmaceutical products. The virus titre was quantified by determining the TCID_50_/mL.

#### Virus inactivation test

The effectiveness of the SARS-CoV-2 inactivation process was evaluated by detecting cytopathic effects and performing a kinetic analysis of subgenomic viral RNA using RT-qPCR in VERO CCL 81.4 cells after SARS-CoV-2 antigen inoculation. Briefly, aliquots of 0.1 mL of the inactivated virus were incubated with 1E + 05 VERO ATCC CCL-81.4 cells for 1 h at 37 °C, followed by the addition of 1.4 mL of DMEM supplemented with 10% FBS. The plates were incubated for 72 h, and in the absence of cytopathic effects, a 0.1 mL aliquot of the supernatant was transferred to a new culture of VERO ATCC CCL-81.4 cells for the second passage. The procedure was repeated at least one more time. For the kinetic analysis of subgenomic viral RNA, 1 mL of inactivated virus was inoculated in 0.1 mL aliquots in ten wells of a 12-well plate containing VERO ATCC CCL-81.4 cells and incubated for 72 h. The supernatant of all wells was pooled, and aliquots of 0.1 mL were placed in a new well of a 12-well plate for the second passage in VERO cells.

For the analysis of increasing amounts of subgenomic RNA during incubation, which indicates virus replication, samples were collected after 1 h, 24 h and 48 h of incubation by lysing the cell monolayer with lysis buffer from the PureLink™ RNA Mini Kit (Thermo Fisher Scientific) according to the manufacturer’s instructions. RNA was extracted, and 100 ng of total RNA were subjected to reverse transcription followed by qPCR using High-Capacity cDNA Reverse Transcription and Fast SYBR™ Green Master Mix kits, respectively (Thermo Fisher Scientific). Quantitative PCR was performed using serial dilutions of a synthetic sequence corresponding to the fragment of amplification as a standard curve (Gene Blocks, IDT). Primers were synthesized based on published methodology^12^.

#### Characterization of the antigen

The purity of the inactivated virus samples was evaluated by performing SDS-PAGE using 4–15% Criterion™ TGX™ Precast Midi Protein gels under nonreducing conditions (Bio-Rad). Electrophoresis was performed for 1 h at 100 V, and the gel was stained using a Pierce™ Silver Stain Kit (Thermo Fisher Scientific). In addition, the amount of residual protein from VERO ATCC CCL-81.4 cells was quantified using the Immunoenzymetric Assay of VERO Cell Host Cell Proteins (Cygnus Technology), as recommended by the manufacturer. The endotoxin content in the purified and inactivated virus samples was quantified using the PYROGENT™ Plus Gel Clot LAL Assay (Lonza, USA) according to the manufacturers’ recommendations. Finally, the purity and identity of the antigen were also evaluated through mass spectrometry analysis as described below.

The antigen quality was analysed based on antigen preservation after the inactivation process using Western blotting. For this analysis, 400 µL of the purified and inactivated antigen were separated on SDS-PAGE gels as described above, and proteins were transferred to a nitrocellulose membrane for 1 h at 90 V—150 mA current. The membrane was blocked with the blocking solution for 3 h at room temperature under agitation. Then, the membrane was washed with phosphate buffer containing 0.05% Tween 20 (3 × 5 min), followed by an incubation with rabbit monoclonal [CR3022] anti-SARS-CoV-2 Spike Glycoprotein S1 antibody (Abcam—Ab273074) and rabbit polyclonal anti-SARS-CoV-2N protein antibody, which was kindly provided by Dr Roxane Piazza (Butantan Institute, Brazil), diluted in sample diluent for 1 h. Peroxidase labelled goat anti-rabbit IgG (KPL) was used for labelling, and the membrane was revealed with SuperSignal West Pico chemiluminescent substrate (Thermo Fisher Scientific). Images were acquired with Uvitec Alliance 2.7 equipment.

#### Immunogenicity analysis of the inactivated virus

The use of mice in the present study was approved by the Institutional Animal Care and Use Committee of the Butantan Institute (n° 9165080620). BALB/c female mice (18–22 g) were obtained from the Center for Animal Breeding of the Butantan Institute. Animals (n = 5) were immunized subcutaneously with purified and inactivated SARS-CoV-2 (corresponding to 1.74 × 10E + 04 TCID_50_/animal) mixed with Al(OH)_3_ (0.25 mg/animal) to achieve a final volume of 200 µL. These animals received three booster doses (1.74 × 10E + 04 TCID_50_/animal) at 7-day intervals. Control animals (n = 5) were inoculated with sterile saline containing Al(OH)_3_ (0.25 mg/animal). Blood was collected 21 and 35 days after priming for serum collection, and samples were stored at − 20 °C until use. Serum samples were further analysed using ELISA as described below and a cytopathic effect-based virus neutralization test (CPE-VNT, as described below). For CPE-VNT, the sera were previously inactivated at 56 °C for 45 min.

### Production of anti-SARS-CoV-2 equine immunoglobulin

#### Animals

The procedures involving horses were approved by the Institutional Animal Care and Use Committee of the Butantan Institute (n° 6016030720).

Ten healthy horses (Creole race) that had not been previously immunized were selected and trained to be manageable for the work. Clinical conditions and behaviours were evaluated, and those horses that were suitable had blood samples collected for laboratory tests, including a complete blood cell count (red blood cells, white blood cells, platelets, haemoglobin, and haematocrit), chemistry panel (total plasma protein, albumin, gamma globulin, alkaline phosphatase, alanine aminotransferase, aspartate aminotransferase, bilirubin, calcium, glucose, sodium, potassium, blood urea nitrogen, and creatinine levels), lipid panel (high-density lipoprotein and low-density lipoprotein levels), creatine phosphokinase levels, and coagulation tests (prothrombin time test and activated thromboplastin time test). Blood samples were collected from the jugular vein and analysed in a veterinary clinical analysis laboratory.

#### Immunization of horses

Horses (n = 10) were subcutaneously injected four times with the inactivated SARS-CoV-2 suspension (corresponding to 4.5 × 10E + 05 TCID_50_/animal/dose) at 1-week intervals (days 0, 7, 14, and 21). Montanide™ ISA 50 V oil-in-water adjuvant (Seppic; Shangai, China) was included in the first and third doses to enhance the antibody response. Serum samples were collected before (day 0), during (day 14), and after (days 28, 35, and 49) immunization (Supplementary Fig. S1) and stored at − 20 °C until use. Haematological and biochemical parameters and antibody titres against SARS-CoV-2 proteins (determined using ELISA and CPE-VNT) were monitored before and after the immunization schedule.

#### Horse plasma collection

Plasma was collected by automated plasmapheresis using a Cobe Spectra^®^ (Terumo BCT) system for therapeutic apheresis procedures. Briefly, a double-lumen catheter was inserted in the jugular vein. One lumen allowed whole blood to be drawn from the horse to which ACD anticoagulant was added. Blood components were separated based on the specific gravity of cells using continuous flow centrifugal technology. Plasma was collected in a transient bag and transferred to a customized bag. The other lumen of the catheter allowed the return of uncollected components to the animal with Ringer’s lactate solution. The plasmapheresis procedure was performed three times (Days 28, 35 and 49 after first dose), according to Supplementary Fig. S1. Each plasmapheresis procedure generated one hyperimmune plasma pool; thus, three pools were obtained sequentially. Plasma samples were analysed for bioburden testing and antibody titres against SARS-CoV-2. Samples from animals were investigated for the presence of SARS-CoV-2 and equine viral encephalitis [Eastern Equine Encephalitis (EEE), Western Equine Encephalitis (WEE), Venezuelan Equine Encephalitis] and equine herpesvirus using PCR in an Equine Veterinary Diagnostic Laboratory to ensure a minimal risk of virus contamination in the starting raw material (plasma).

#### Purification of immunoglobulins from horse plasma

The starting plasma pool was subjected to fractionation by precipitation with ammonium sulphate. The precipitate was centrifuged, and the liquid fraction was discharged. The product was then solubilized, and the pH was adjusted for enzymatic digestion with pepsin to hydrolyse the non-IgG proteins and cleave the molecule into F(ab′)_2_ fragments, removing the Fc fragment. A second precipitation was performed by adding ammonium sulphate, and the pH was adjusted by adding caprylic acid with stirring. Next, thermocoagulation at 56 °C separated nonspecific thermolabile proteins, whereas the precipitate was eliminated. The supernatant was subjected to diafiltration and ion-exchange chromatography and concentrated through tangential ultrafiltration. Phenol was added as a preservative, and the chloride concentration was adjusted. The F(ab′)_2_ solution was then subjected to 0.22 µm filtration and maintained in disposable plastic bags. All production steps were performed under GMP (good manufacturing practices) conditions.

#### Protein profile of the final product (batches 00001, 00002, and 00003)

The protein profiles of the batches of final products (equine anti-SARS-CoV-2 F(ab′)_2_ immunoglobulin) were analysed using SDS-PAGE (12.5%) under reducing conditions. Samples of each batch were incubated at 37 °C for 30 min, 1 h, 2 h or 3 h and separated on SDS-PAGE gels to evaluate the presence of any proteolytic product. Gels were stained with Coomassie Blue dye (40% methanol, 10% acetic acid and 0.25% Coomassie blue) and destained with 40% methanol containing 10% acetic acid.

#### Protein identification using LC–MS/MS analysis (liquid chromatography and tandem mass spectrometry)

Samples of the purified inactivated virus (batch 8911/20) and from the equine anti-SARS-CoV-2 F(ab′)_2_ immunoglobulin (batches 00001, 00002, and 00003) were subjected to the FASP protocol^13^ using 10 kDa filter units (Merck-Millipore). Tryptic peptides were desalted using homemade stage tips, and the eluted peptides were dried under a vacuum using a centrifugal concentrator (Eppendorf). Samples were dissolved in 20 µL of aqueous buffer containing 0.1% formic acid, and 2 µL of each sample were injected into an Acclaim PepMap100 C18 trap column (Thermo Fisher Scientific) with a 3 μm particle size, 100 Å pore size, 75 μm internal diameter, and 20 mm column length and analysed using an Orbitrap Q-Exactive plus (Thermo Fisher Scientific) mass spectrometer coupled to an Easy nanoLC 1200 (Thermo Fisher Scientific) at a flow rate of 200 nL/min. Buffer A contained 0.1% (v/v) formic acid, and buffer B contained 0.1% (v/v) formic acid in 80% acetonitrile. Peptides were applied with an increasing gradient of acetonitrile over time (0–90% (v/v) acetonitrile in 30 min) to separate the peptides on an analytical Acclaim PepMap column (Thermo Scientific) with a 2 µm particle size, 100 Å pore size, length of 150 mm, and inner diameter of 50 µm. The spray voltage was set to 2.4 kV, and the mass spectrometer was operated in positive, data-dependent mode, in which one full MS scan was acquired in the *m/z* range of 300–1500 followed by MS/MS acquisition using higher-energy collisional dissociation (HCD) of the seven most intense ions from the MS scan using an isolation window of 2.0 *m/z*.

#### LC–MS/MS data analysis

The obtained MS and MS/MS spectra were analysed using PEAKS STUDIO version X, and the searches were performed against a customized database. Briefly, for antigen data analysis, the reference bank used included all SARS-CoV-2 sequences and reviewed *Chlorocebus aethiops* sequences downloaded from UniProt (total of 162 sequences, downloaded on 10-01-2020). For the analysis of the three batches of equine anti-SARS-CoV-2 F(ab′)_2_ immunoglobulin, the reference bank was constructed using all anti-SARS-CoV-2 sequences downloaded from GenBank, and reviewed *Equus caballus* sequences from UniProt (total of 2190 sequences downloaded on 10-09-2020). Both reference databases were concatenated with common contaminants for mass spectrometry experiments (116 sequences), and the decoy sequences were used for false discovery rate control. The search engine was set to detect semitryptic peptides at an FDR (false discovery rate) of 0.01. Methionine oxidation and acetylation of the protein N-termini were set as variable modifications, and carbamidomethylation of cysteine was set as a fixed modification. Features identified as contaminants and proteins without unique peptides were excluded. Spectral counts were used to estimate the protein proportion in each sample.

### Anti-SARS-CoV-2 serum potency analyses

#### ELISA for anti-SARS-CoV-2 IgG detection

ELISA plates (Costar^®^) were coated with 100 µL of purified and inactivated SARS-CoV-2 (1 × 10E + 03 virus/mL or 2 × 10E + 03 virus/mL for mouse and horse, respectively) and incubated overnight at 4 °C to evaluate the serum antibody titres of the immunized mice and horses. The plates were then blocked with 5% BSA in PBS for 2 h at 37 °C and incubated with serial dilutions of nonimmune or experimental serum samples for 1 h at 37 °C. After incubation, the plates were washed with PBS containing 0.05% Tween 20 and incubated with anti-mouse IgG-HRPO (1:2000, Zymed) or anti-horse IgG-HRPO antibodies (1:5000, Jackson ImmunoResearch Lab) in 0.1% BSA/PBS-Tween 20 0.05% for 1 h at 37 °C. The plates were then washed, and the reactions were developed with TMB substrate (BD OptEIA™) according to the manufacturer's instructions. Absorbance values were recorded in an ELISA reader (BioTek Elx800 spectrophotometer) at 450 nm. The titre was established as the highest serum dilution in which the measured absorbance was twice as high as that determined for nonimmunized mouse serum (control group) or for preimmune horse plasma.

#### Cytopathic effect-based virus neutralization test (CPE-VNT)

CPE-VNT was carried out using SARS-CoV-2 wild-type variant B.1.1.28 (SARS-CoV-2/human/BRA/SP02/2020; MT126808.1) to evaluate the plasma neutralizing antibody titres of the immunized mice and horses. The 3 batches of the final product (equine anti-SARS-CoV-2 serum) were also tested against the P.1./Gamma SARS-CoV-2 variant (IMT 87201 strain) and P.2./Zeta variant (hCoV-19/Brazil/RS-00601/2020-LMM-EPI_ISL_779155, which were kindly provided by Dr. Fernando Spilki—Feevale University, RS, BR. The assays were performed using 96-well plates seeded with 2 × 10E + 04 VERO ATCC CCL-81.4 cells per well, 24 to 28 h prior to the experiment. A serial dilution of each serum (1:20 to 1:40,960) in DMEM supplemented with 2.5% FBS was performed, and then 100 TCID_50_ of the virus (v/v) was added. The virus/serum mixture was incubated at 37 °C for 1 h to allow virus neutralization. Thereafter, the mixture was transferred onto the confluent VERO ATCC CCL-81.4 cell monolayer and incubated for 72 h at 37 °C with 5% CO_2_. After the incubation period, the plates were microscopically inspected for CPE caused by SARS-CoV-2 and subsequently stained with 0.2% naphthol blue black solution and analysed to confirm the titre. The virus neutralization titre, referred to as VNT_100,_ is described as the highest dilution of serum that neutralizes virus growth. In each assay, a positive serum and a negative serum were included as controls.

#### ELISA kit cPass™

The cPass™ SARS-CoV-2 Neutralization Antibody Detection Kit (GenScript, Cat.: L00847) was used according to the manufacturer’s protocol for qualitative direct detection of total neutralizing antibodies to SARS-CoV-2 in equine and hamster sera^14^. For the three batches of equine anti-SARS-CoV-2, samples were diluted 1:100, 1:1000 and 1:2500 with PBS, pH 7.4. For the hamster serum, samples were diluted 1:10, 1:20 or 1:40 with PBS, pH 7.4. The serum neutralization plate was blocked with 200 μL/well of PBS + BSA 1% and then incubated at 37 ± 2 °C for 1 to 2 h. Samples and controls were added to the wells and incubated at 37 ± 2 °C for 30 min with the anti-SARS-CoV-2 RBD-HRP solution. The same samples and controls were then transferred from serum neutralization plates to capture plates (ELISAs), incubated at 37 ± 2 °C for 15 min and washed four times with a 1 × wash solution (1:20 dilution with ddH_2_O, 300 µL/well). After the washing step, the TMB substrate solution was added (100 µL/well) and incubated at ambient temperature (25 ± 3 °C) for 20 min in the dark. Finally, the provided stop solution was added (50 µL/well) to interrupt the HRP and TMB reaction. The absorbance of the samples was measured immediately at 450 nm. Competitive ELISA was performed under GLP conditions, and the methodology was previously validated for usage with equine serum.

#### Elecsys^®^Anti-SARS-CoV-2 S

The three batches of equine anti-SARS-CoV-2 F(ab′)_2_ immunoglobulin were diluted in sterile pyrogenic saline solution at a ratio of 2 from 1/20 to 1/20,480. The samples were transferred to collection tubes of the vacutainer type compatible with Cobas and Roche equipment. The analyses were performed in an automated manner using the Elecsys^®^ Anti-SARS-CoV-2 kit (Roche Diagnostics International AG) for the quantitative determination of antibodies (including IgG) against the RBD domain of the SARS-CoV-2 spike protein. The results are reported as U/mL based on the kit's standard monoclonal antibody curve.

### Challenge test in Golden Syrian hamsters

#### Animals

The procedures described here were approved by the Institutional Animal Care and Use Committee of Butantan Institute (n°9165080620) and the Institute of Biomedical Sciences of the University of São Paulo (n° 6103261120).

#### Study design

Seventy-two conventional Golden Syrian hamsters (*Mesocricetus auratus*) (36 males and 36 females, 115–148 g, 10–11 weeks old) were obtained from the Center for Animal Breeding of Butantan Institute. Experiments were performed in a biosafety level 3 animal facility. Hamsters were housed individually and divided into four groups of 18 animals each (9 males and 9 males), normalized based on weight. On Day zero, animals were anaesthetized with ketamine hydrochloride (100 mg/kg) and xylazine hydrochloride (7 mg/kg), and two groups (G1 and G2) were inoculated intranasally with 50 μL of 1 × 10E + 05TCID_50_ of SARS-CoV-2 (SARS-CoV-2/SP02/2020HIAE, GenBank MT12680.1, 4th passage in VERO ATC-CCL81.4 cells) in DMEM with 2% FBS, while the remaining two groups (G3 and G4) were inoculated with 50 μL of DMEM containing 2% FBS. On Day 2 p.i. (postinfection), hamsters of G1 and G3 were intraperitoneally injected with 500 µL of equine anti-SARS-CoV-2 F(ab′)_2_ immunoglobulin (7.2 mg/animal, equivalent to ~ 3365 U/RBD/animal), while hamsters of G2 and G4 received 500 μL of sterile apyrogenic saline solution via intraperitoneal injection. On Days 3, 5 and 7 p.i., subgroups of 6 animals (3 males and 3 females) from each group were euthanized with overdoses of ketamine hydrochloride (600 mg/kg) and xylazine hydrochloride (30 mg/kg) and necropsied. Nasal turbinates, trachea, and fragments of the lungs were separately collected in lysis buffer (Mag MaxCore kit, Thermo Fisher Scientific) (for RNA extraction), in viral transportation medium [VTM − DMEM + 2% FBS + antibiotics/antifungal (Vitrocell − penicillin 10.000U, streptomycin 10 mg, 25 μg de amphotericin B/mL] (for virus titration), and in 10% formalin (for histopathology analysis). All five lobes of the lungs were represented in each sample for RNA extraction and virus isolation and were analysed separately using histopathology. Whole lungs and all tissue fragments were weighed. Samples collected for virus isolation were quickly frozen in liquid nitrogen and stored at − 80 °C until processing. The animals were also bled, and the serum was analysed for the presence of horse antibodies using capture ELISA and the ELISA kit cPass™.

#### Viral load quantification from tissue samples

The viral load was quantified by determining the TCID_50_/g of tissue (using samples in VTM) and the number of viral RNA copies/β actin RNA copies/gram of tissue (using samples in lysis buffer). Briefly, all samples of nasal turbinates, trachea, and lungs were thawed and subjected to disruption using 2 mm glass beads in TissueLyser II equipment (Qiagen) at 30 Hz for 2 min twice, followed by centrifugation at 13,000 rpm (Eppendorf 5804R centrifuge) for 30 s. The supernatants were used for viral load quantification. The TCID_50_ determination was performed as described above.

Total nucleic acids were extracted using a Magmax Core Kit with a MagMAX Express Magnetic Particle Processor (Thermo Fisher Scientific) according to the manufacturer’s instructions. The detection of SARS-CoV-2 RNA was performed based on a previously described protocol^9^ using a one-step real-time quantitative PCR (qRT-PCR) assay kit (AgPath-ID™ One-Step RT-PCR Reagents, Applied Biosystems Inc.) and an ABI 7500 SDS real-time PCR machine (Applied Biosystems). The duplex reaction was performed using specific primers for SARS-CoV-2 and primers for hamster β actin (ActB Rv 5′ CAC CAT CAC CAG AGT CCA TCA C 3′, ActB F CTG AAC CCC AAA GCC AAC; ActB_P- HEX TGT CCC TGT ATG CCT CTG GTC GTA ZEN/IOWA BLACK) as a housekeeping gene. The number of RNA copies/mL was quantified based on a standard curve obtained by serially diluting a synthetic dsDNA sequence (Gene Blocks, IDT) corresponding to the amplification fragment of the target gene.

#### Capture ELISA for the detection of horse antibodies in hamster serum samples

Capture ELISAs were performed to identify horse antibodies in hamster serum samples. Briefly, ELISA plates were coated with 100 µL of rabbit anti-horse IgG (500 ng/mL) (Sigma) overnight at 4 °C. The plates were washed with 200 µL of PBS and blocked with 200 µL of BSA 5% in PBS for 2 h at 37 °C. The plates were then washed three times with 200 µL of PBS, and the previously prepared standard curve (anti-SARS-CoV-2 horse F(ab′)_2_) immunoglobulin serially diluted in PBS, from 262 µg/mL to 0.13 µg/mL) and serum samples (diluted 1:10 in PBS) were added in triplicate. After 1 h of incubation at 37 °C, the plates were washed three times with PBS containing 0.05% Tween 20 and incubated with peroxidase-conjugated anti-horse IgG (diluted 1:5000 in BS with 0.05% Tween 20 and 0.1% BSA) (Jackson ImmunoResearch Lab) for 1 h at 37 °C. The plates were washed three times with PBS containing 0.05% Tween 20, and the reactions were developed for 15 min by an incubation with TMB substrate (BD OptEIA™) according to the manufacturer's instructions. Absorbance values were recorded using an ELISA reader (BioTek Elx800 spectrophotometer) at 450 nm. The standard curve was linearized by log–log transformation and then subjected to linear regression analysis. The concentration of horse F(ab′)_2_ in serum samples was estimated by interpolating the samples to the standard curve.

#### Histopathological examination

Lung samples preserved in 10% formalin were subjected to routine fixation and paraffin embedding, cut and stained with haematoxylin and eosin (HE). Histopathological findings of changes related to SARS-CoV-2 infection were assessed as described in the literature^15–17^. Tissue samples were blindly evaluated by certified veterinary pathologists and examined under a light microscope for the presence of cellular/tissue alterations in the lung, as described in detail in Table S1 (Supplementary Material). Five anatomical structures of the lungs were analysed per animal and identified as the right cranial lobe, right middle lobe, right caudal lobe, accessory lobe and left lung. Histopathological scores were established as very light (I), light (II), moderate (III), and severe (IV). Using this metric, we were able to quantify the degree of bronchitis, oedema, and pneumonia^18,19^.

#### Analysis of leukocyte infiltration in the lung tissue

The numbers of neutrophils, monocytes and lymphocytes present in the hamster lung tissue were determined to better analyse the inflammatory process. For this experiment, images of five fields of H&E-stained sections from each animal were captured under a microscope (Nikon Eclipse Ti-S) using a digital camera (DS-Fi1c, Nikon). For leukocyte counting, ImageJ software and the Colour Deconvolution 2 plugin were used to visualize and separate nuclei from the cytoplasm (Fig. S2—Supplementary Material)^20^. For cell counting, the Cell Counter plugin was used. This analysis facilitated the differential counting of segmented and mononuclear nuclei.

#### Analysis of cellularity and functional area in the lung tissue

Cellularity and functional lung area represented by the intra-veolar regions in hamster lung tissue were determined conformed^21^. For this experiment, five H&E stained sections of each lung lobe per animal were captured under a microscope (Nikon Eclipse Ti-S) using a digital camera (DS-Fi1c, Nikon). To measure the areas of interest, ImageJ software and the Color Deconvolution 2 plugin were used to visualize and separate the cytoplasmic nuclei. The images were transformed into 8-bit and treated with threshold and percentage of the measured area.

#### Immunofluorescence staining of lung tissue

Thin sections of lung tissue were subjected to deparaffinization, rehydration and antigen recovery. Endogenous peroxidases were then blocked, followed by tissue permeabilization using 0.4% Triton X-100 in PBS. Nonspecific binding was blocked, and the thin sections were washed twice with PBS containing 0.05% Tween 20 and incubated with anti-SARS-CoV-2 spike glycoprotein rabbit antibody (Abcam, ab272504, 1:100) for one hour at room temperature. The thin sections were then washed with PBS containing 0.05% Tween 20 twice and incubated with the Alexa Fluor 647-conjugated anti-rabbit antibody (Thermo Fisher Scientific; A21244, 1:100) for 30 min in a dark chamber at room temperature. After washing, anti-horse IgG FITC (Sigma-Aldrich; F-7759, 1:100) was added and incubated for one hour at room temperature. The thin sections were washed, and the slides were mounted using Hoechst (Thermo Fisher Scientific; 62249, 1:1000) and ProLong™ Glass Antifade Mountant (Thermo Fisher Scientific; P36980). The sections were analysed under a confocal microscope (Leica TSC SP8 DSL Hyvolution). The images were assembled and analysed in 3D using Imaris Viewer software.

### Statistical analysis

Data are presented as the means ± standard errors and were statistically analysed with GraphPad Prism version 9.1 software for Windows (San Diego, CA, USA). For comparisons, the following tests were used: one-way ANOVA followed by Tukey’s posttest or Student’s t-test followed by the Mann–Whitney test.

### Ethics declarations

All methods were carried out in accordance with the relevant guidelines and regulations. All animal experiments comply with the ARRIVE guidelines.

## Results

### Antigen production

SARS-CoV-2 was propagated in 6 L batches using static monolayer VERO ATCC-CCL81.4 cells cultured in serum-free medium. The virus was efficiently harvested and clarified by depth filtration with 100% recovery. The active virus showed good stability at 4 °C for at least 24 h under this condition. Concentration by TFF using a 300 kDa membrane also resulted in good recovery yields (> 90%), and no virus was actually be quantified in the permeate. Two-step ultracentrifugation was applied for virus purification. After the first step, the virus was recovered in many fractions of the sucrose gradient, which were then pooled, diluted and loaded on the second gradient. The final purified material was recovered from a narrower gradient fraction (58 to 35% sucrose). Virus inactivation by 15 kGy gamma irradiation was effective and safe, considering a sterility assurance level (SAL) applied for materials that are intended to be in contact with humans and animals. Inactivation tests showed no cytopathic effect on VERO ATCC-CCL81.4 cell monolayers after three blinded passages. Tests for viral subgenomic RNA kinetics also showed no evidence of viral replication in VERO ATCC-CCL81.4 cells after 72 h of incubation, as no significant differences in the RNA level were detected over the kinetic evaluation (data not shown).

### Antigen analysis

**Figure 1:**
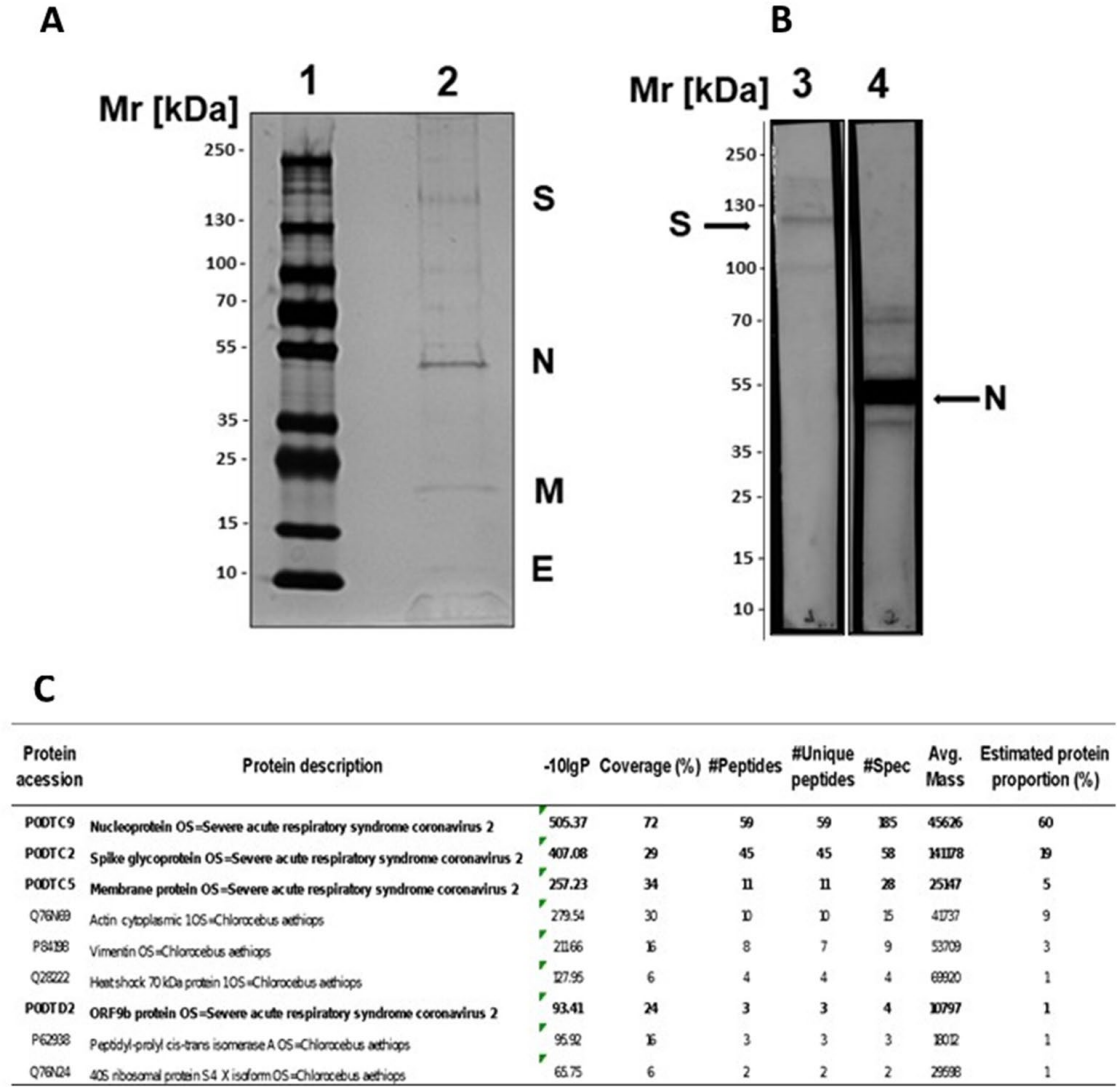
Analysis of the purified and inactivated SARS-CoV-2. (**A**) SDS–PAGE: 4–15% gradient SDS–PAGE gel under nonreducing conditions and silver staining. (1) LMW marker—10 to 250 kDa; (2) Purified and inactivated SARS-CoV-2. (**B**) Western blot: samples of the virus, which were separated on 4–15% gradient SDS-PAGE gels, were blotted onto nitrocellulose membranes and incubated with monoclonal antibodies against the SARS-CoV-2 S protein (Lane 3) or anti-SARS-CoV-2N protein (Lane 4). The membranes were incubated with specific peroxidase-conjugated anti-rabbit IgG (1:5000), and the reactions were revealed using SuperSignal West Pico chemiluminescent substrate. (**C**) Protein identification through tandem mass spectrometry analysis (LC–MS/MS) of the inactivated SARS-CoV-2 purified antigen. Only proteins with at least 1 unique peptide, a score − log10P > 20 and a false discovery rate < 1% were accepted for identification and are depicted in the Table. The proportion of proteins was estimated based on the mass spectra counts of each identified protein. (**A**) and (**B**) were cropped, the original results are presented in Fig. S7. (**B**) is composed of two panels, representing two membrane strips processed separately, and a third strip containing a molecular weight marker was used do estimate the molecular sizes of bands (Fig. S7).

The purity of the antigen was verified by SDS-PAGE (Fig. 1A), and four main fragments with sizes of the four viral structural proteins (N, M, E and S) were detected^22^. In addition, other fragments were verified in lower concentration that could represent oligomers of the S and N proteins^23–25^. Other proteins are probably residual protein content of VERO ATCC-CCL81.4 cells (HCP—host cell protein) which was measured using a specific ELISA and found to be 234.33 ng/mL.

Western blots using specific antibodies against the SARS-CoV-2 S protein and anti-SARS-CoV-2N protein recognized different forms of both proteins in the purified and inactivated viruses (Fig. 1B). In addition, these three viral proteins (N, S and M) were identified by performing an LC–MS/MS analysis of the antigen (Fig. 1C). Furthermore, the protein content proportion estimated based on spectral counts showed that total viral proteins represented approximately 90% of the purified and inactivated antigen samples (Fig. 1C). Endotoxin measurements in the purified and inactivated virus samples showed a level below the sensitivity of the assay (< 0.125 EU/mL) (data not shown).

**Figure 2:**
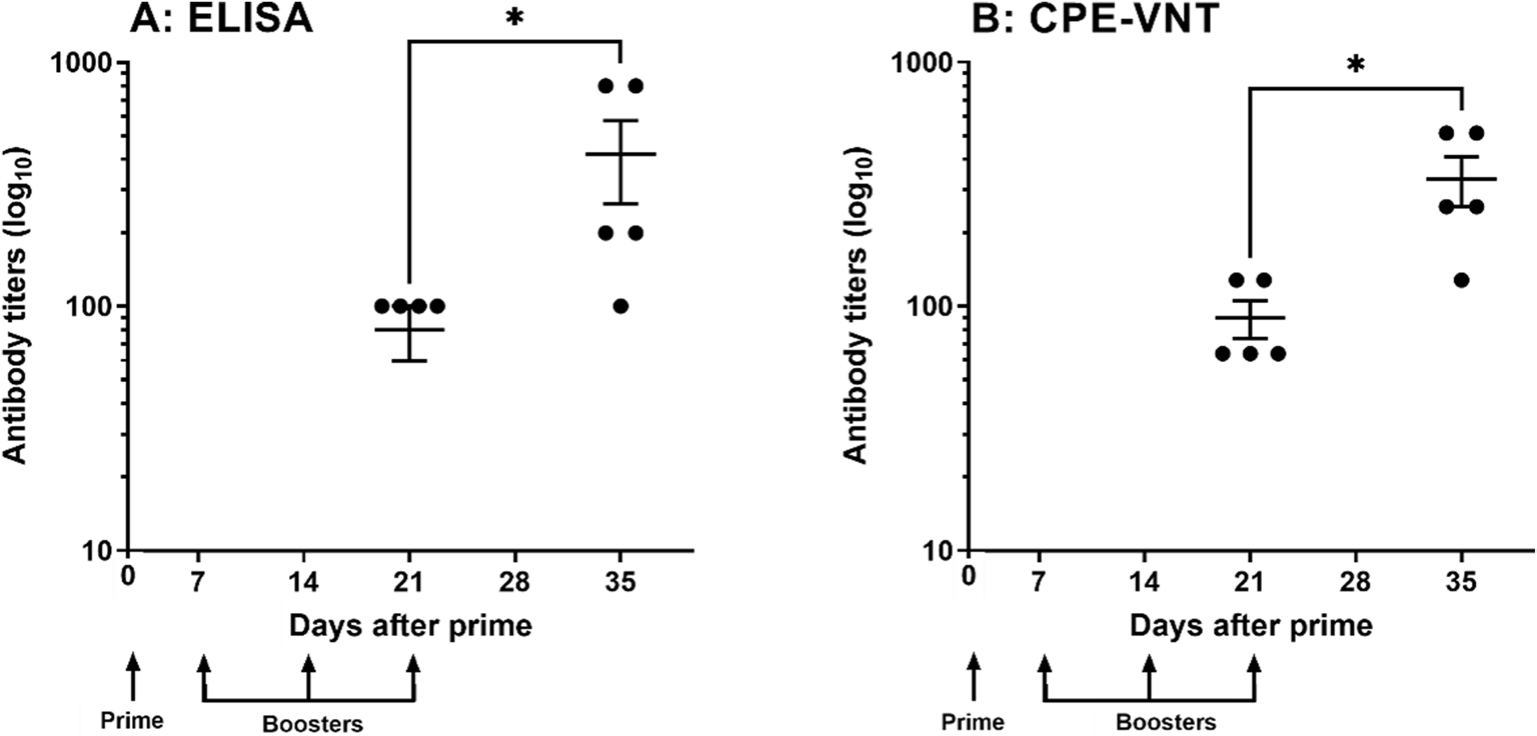
Purified and inactivated SARS-CoV-2 virus immunogenicity in mice. (**A**) ELISA plates were coated with 100 µL of purified and inactivated SARS-CoV-2 (1 × 10E + 03 virus/mL), incubated with increasing dilutions of nonimmune or experimental sera obtained from BALB/c mice, and incubated with anti-mouse HRPO-conjugated IgG. The reaction was performed by adding TMB substrate, and the absorbance was detected at λ 450 nm using a spectrophotometer. The titre was established as the highest serum dilution at which the measured absorbance was twice as high as that determined for nonimmunized mouse serum (control group). (**B**) Sera from mice immunized with purified and inactivated SARS-CoV-2 antigen were also tested using the CPE-VNT. The neutralization titre was defined as the inverse of the highest dilution of serum that blocks viral replication. Statistical analyses were performed using Student’s t-test followed by the Mann–Whitney test (*p ≤ 0.05).

The immunogenicity of the purified and inactivated virus samples, as well as their potential toxicity in vivo, were evaluated by measuring the specific antibody response elicited after the immunization of mice and by determining toxicity parameters, respectively. As shown in Fig. 2A, the inactivated virus was immunogenic and induced increased production of antibodies over time after immunization, as measured using an ELISA with the virus serving as the antigen. The presence of neutralizing antibodies against live SARS-CoV-2 (variant B.1.1.28) was also measured. The average titres of neutralizing antibodies in the mice, as determined using CPE-VNT, after four immunizations with SARS-CoV-2 antigen were 1:256 (Fig. 2B). Moreover, the repeated inoculation of the inactivated virus in the murine model did not produce any toxic effect on the animals (data not shown). Thus, these results allow us to use purified and inactivated viruses for horse immunization.

### Production of anti-SARS-CoV-2 hyperimmune plasma

Horses were subcutaneously injected four times with inactivated SARS-CoV-2 suspension at 1-week intervals. Laboratory tests did not show any relevant alterations in haematological parameters throughout the immunization scheme. The biochemical analysis showed normal values, except for a slight increase in total protein and gamma globulin levels after the administration of the four immunization doses and before the plasmapheresis procedures (Table S2—Supplementary Material).

Serum samples were collected before and during immunization, and antibody titres against SARS-CoV-2 proteins were monitored using ELISA and CPE-VNT. Similar to the observations in mice, the titration of horse plasma samples verified the effectiveness of the immunization schedule, as the highest antibody titres 28 days were observed after the primary immunization (Fig. S3A—Supplementary Material). As expected, horses developed much higher antibody titres to inactivated SARS-CoV-2 than mice, resulting in three hyperimmune plasma pools for the production of F(ab′)_2_ immunoglobulin fragments on Days 28, 35 and 49 after the primary immunization. Moreover, increased levels of neutralizing antibodies were also observed in horses during the immunization process (Fig. S3B—Supplementary Material).

Plasma was collected from the horses when anti-SARS-CoV-2 antibody levels achieved the highest peak and after animals were examined by a veterinarian and considered healthy. Animals were subjected to automated plasmapheresis, and veterinary staff monitored the animals throughout plasma collection. The procedure lasted an average of 3 h, a period in which animals were fed and water was offered, until at least 10–12 L of plasma were collected, or animals presented any signs of distress due to permanence in the stalls. After automated plasma collection, a tendency towards a slight decrease in the serum total protein and gamma globulin levels was observed between the 2nd and the 3rd plasmapheresis. Basal values for weight and serum protein levels occurred at most seven days after the first two plasmapheresis procedures, except for the third plasma collection. In this case, we opted to delay for an additional week to allow the recovery of serum protein levels (Table S2—Supplementary Material).

Three pools of hyperimmune plasma were obtained, and the final volume of each pool was composed of the sum of the plasma collected from the 10 animals at different time points during the immunization schedule. The parameters of the plasma collection process are shown in Table S3 (Supplementary Material). One week after automated plasmapheresis, parameters recovered to normal levels in the animals. Horses were maintained in a paddock with a regular supply of pasture and additional grain and water under veterinarian surveillance.

All samples of animal plasma were negative for SARS-CoV-2 and for viruses that could possibly infect horses and are considered pathogenic to humans, i.e., equine herpesvirus 1–4 and equine encephalitis virus (Eastern, Western and Venezuelan) virus.

### Production of anti-SARS-CoV-2 equine immunoglobulin

A total of 428.130 mL of plasma was collected and submitted for industrial processing. Bulk samples resulted in three batches of purified specific fragmented anti-SARS-CoV-2 immunoglobulin, which were filled in 5.0 mL vials. Samples of the three batches were subjected to SDS-PAGE analysis to evaluate the existence of proteolytic products. No changes in the protein profile of the three batches were observed even after an incubation for 3 h at 37 °C, indicating a lack of proteolytic events in anti-SARS-CoV-2 samples (Fig. S4A). Moreover, the tandem mass spectrometry analysis revealed the presence of specific anti-spike regions of immunoglobulins in samples from the three batches (Fig. S4B).

The samples were tested at the Butantan Institute Quality Control Center for appearance, pH, osmolality, vial sealing, sodium chloride, phenol, ammonium sulphate, nonprotein nitrogen, total solids, total protein, extractable volume, deliverable volume, purity, molecular distribution, protein aggregates, sterility, and pyrogens. All the results were consistent with the Brazilian Pharmacopeia (data not shown). Specifically for phenol, concentration did not exceed 11 mg per vial, which was far under the limit specified (maximum 17.5 mg).

### Analysis of the potency of the equine anti-SARS-CoV-2 F(ab′)_2_ immunoglobulin

The potency of anti-SARS-CoV-2 hyperimmune serum was assessed using three different methodologies: competitive ELISA (GenScript) described as a surrogate virus neutralization test based on antibody-mediated blockage of ACE2-spike protein–protein interaction^14^, CPE-VNT for neutralizing antibody detection and quantitative ELISA for anti-RBD domain antibody detection (ROCHE) (Table 1). All three batches contained neutralizing antibodies when tested using CPE-VNT. The mean neutralizing antibody titres against the three variants currently circulating in Brazil ranged from 1120 to 2140 for the B.1.1.28 variant and from 100 to 200 for P.1. Gamma variant and from 1120 to 2560 for the P.2./Zeta variant (Table 1). These results are consistent with the inhibition evaluated using competitive ELISA, in which all anti-SARS-CoV-2 serum batches analysed were positive (cut off = 20%) for the presence of a neutralizing antibody at least until the dilution 1/2500. On the other hand, quantification of total anti-spike protein antibodies showed that the titre ranged from 5477.6 to 7798.3 U/mL.

**Table 1:** Equine Anti-SARS-CoV-2 F(ab′)_2_ immunoglobulin potency.

	Neutralizing antibody^a^		Anti-spike^b^	Neutralizing antibody^c^		
	Dilution factor	% Inhibition		B.1.1.28	Gamma	Zeta
SCOV200001	1/100	99.1	6727.5 U/mL	1120	200	1440
	1/1000	72.5				
	1/2500	43.1				
SCOV200002	1/100	99.4	7798.3 U/mL	2140	140	2240
	1/1000	76.0				
	1/2500	43.8				
SCOV200003	1/100	99.2	5477.6 U/mL	1280	120	1120
	1/1000	50.9				
	1/2500	28.2				

^a^cPass Neutralization Antibody Detection kit (GenScript #L00847).

^b^Elecsys^®^ Anti-SARS-CoV-2 S (Roche # 09289267190).

^c^VNT-CPE assay using B.1.1.28. Gamma and Zeta strains.

### Hamster infection with SARS-CoV-2

**Figure 3:**
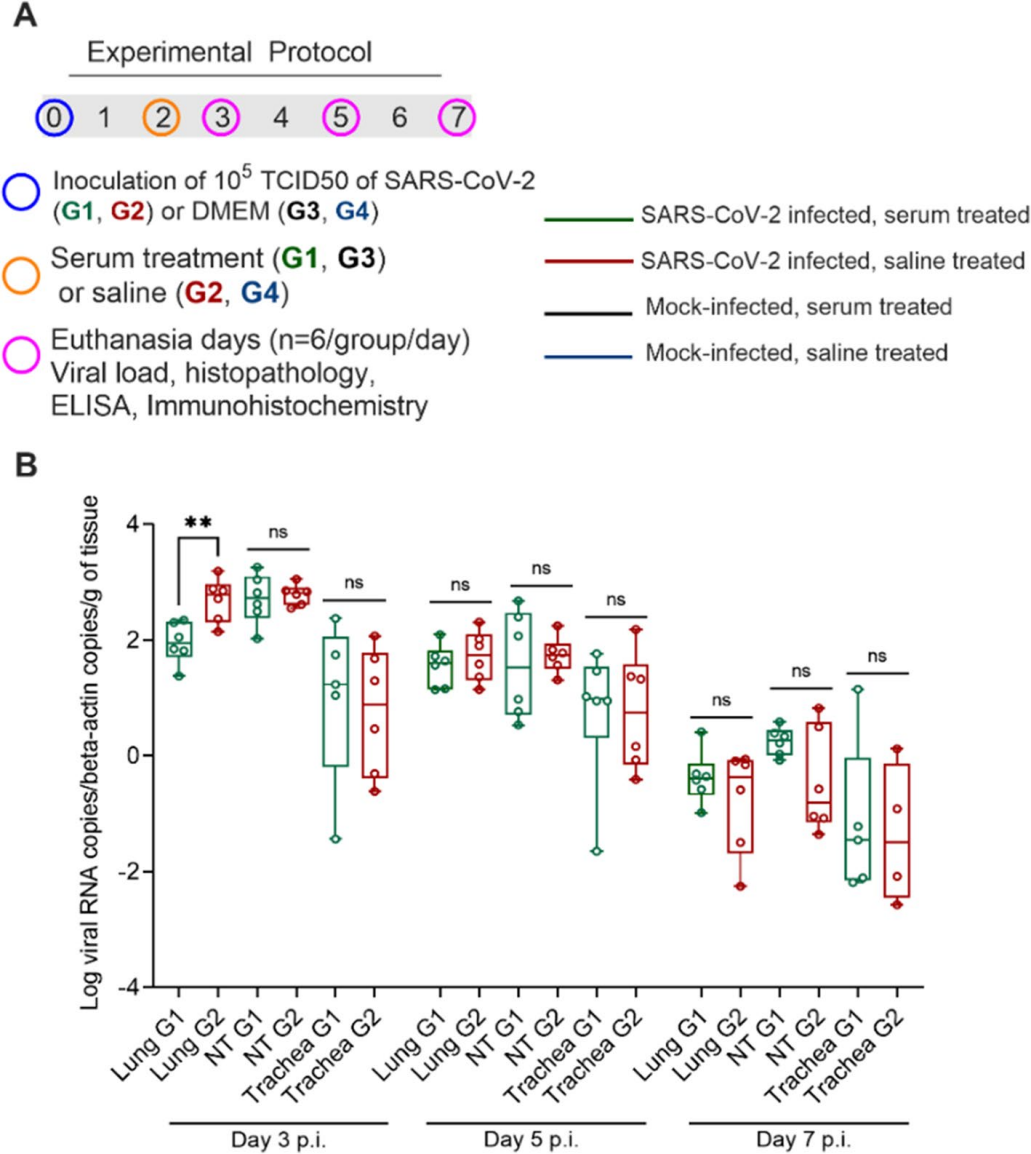
Challenge test in Golden Syrian hamsters. (**A**) Study design—scheme for infecting animals with SARS-CoV-2 followed by treatment with equine anti-SARS-CoV-2 F(ab′)_2_ immunoglobulin. (**B**) SARS-CoV-2 RNA copies as measured using RT-qPCR per number of β-actin RNA copies per gram of tissue. Green: G1 animals (n = 6 per subgroup), i.e., serum-treated, SARS-CoV-2-infected animals; red: G2 animals (n = 6 per subgroup), i.e., nontreated, SARS-CoV-2-infected animals. p.i. = post infection; n.s.: not significant (p value > 0.05); **p value = 0.0043 (Day 3 p.i.).

Golden Syrian hamsters were used for the preclinical evaluation of the protective efficacy of equine anti-SARS-CoV-2 F(ab′)_2_ immunoglobulin. We first evaluated the effectiveness of the treatment administered at 2 Days p.i. by evaluating body weight, clinical signs, and viral load in the lung, trachea and nasal turbinates (Fig. 3A). As expected, SARS-CoV-2-infected animals showed progressive weight loss compared to noninfected animals, with a slight recovery on Days 6 and 7 p.i. Weight changes observed in SARS-CoV-2-infected animals that received serum treatment were not different from those in SARS-CoV-2-infected animals that received saline (p value > 0.05) (Fig. S5A—Supplementary Material). Clinical signs of sneezing and snout rubbing were evident beginning on Day 3 p.i. in all infected animals, persisting until the last day of the experiment, and were absent in noninfected animals.

Although no significant difference was observed in virus isolation in terms of the TCID50 from the respiratory tract between treated and nontreated animals (Fig. S5B—Supplementary Material), SARS-CoV-2-specific RT-qPCR showed a significantly lower viral load in the lungs of serum-treated animals on Day 3 p.i. than in animals that did not receive equine serum (Fig. 3B). Viral loads were all negative in noninfected animals. The individual data for each experimental group are shown in Table S5 in the supplementary material.

### SARS-CoV-2 pathology in the hamster model

On Day 3 p.i., gross pathological lesions compatible with haemorrhage in the lungs of SARS-CoV-2-infected animals (G1 and G2) were observed, progressing to extensive pulmonary involvement (frequently > 50% of lobes) on Days 3, 5 and 7 p.i. On Day 7 p.i., hepatization of lung areas was common in SARS-CoV-2-infected animals. No significant macroscopic lesions were observed in other organs of SARS-CoV-2-infected animals or in the animals not infected with SARS-CoV-2.

**Figure 4:**
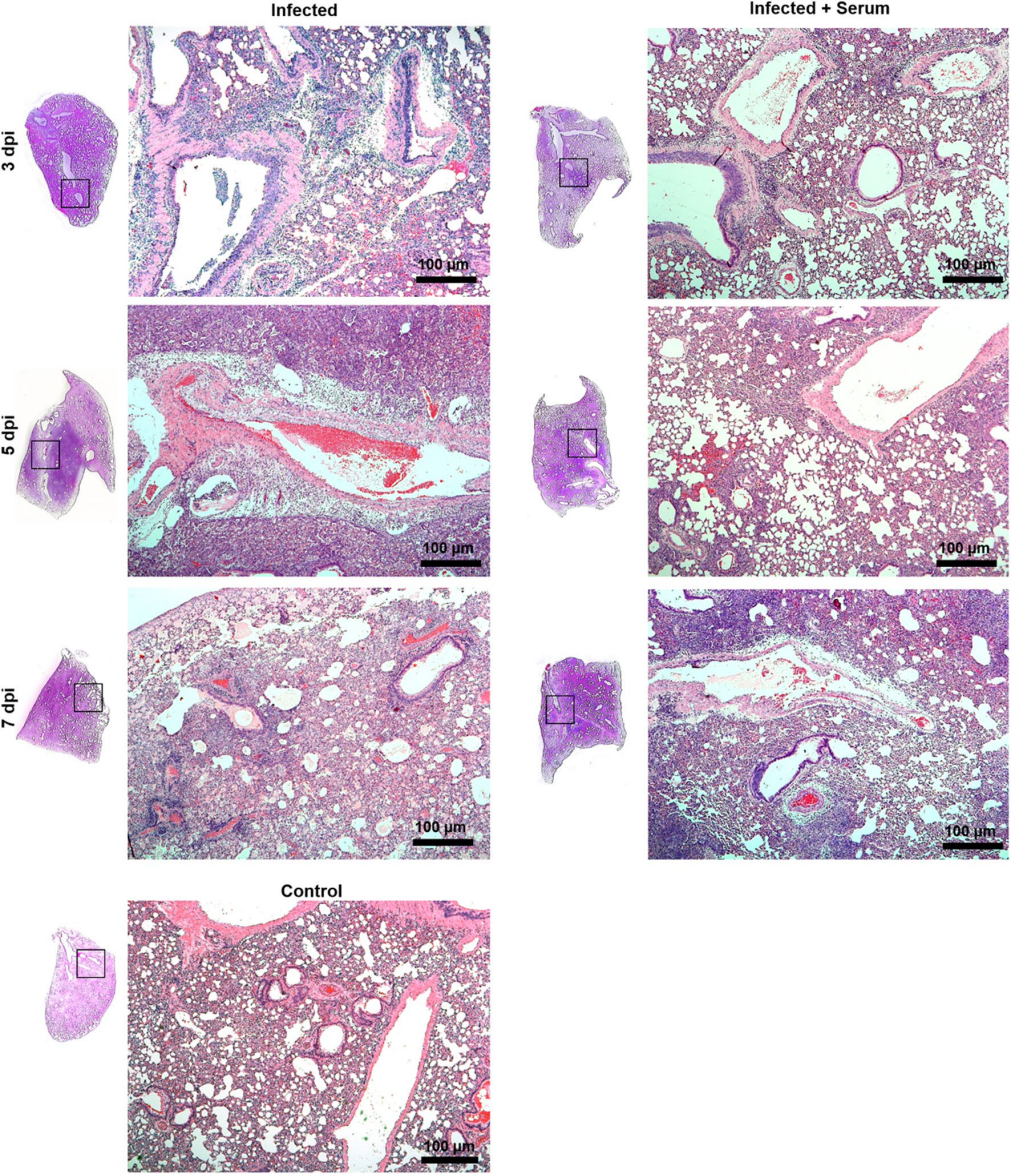
The pneumonic pattern of animals infected and treated with equine anti-SARS-CoV-2 F(ab′)_2_ immunoglobulin. Panels show the evolution of the infectious process in the course of SARS-CoV-2 hamster experimental infection and treatment with equine serum. Infected and nontreated animals showed a more marked evolution of the diffuse interstitial pneumonia pattern, mainly on Day 7 after infection, than infected and SARS-CoV-2-treated animals. Control samples show the normal histological pattern of the tissue. Bar 100 µm; haematoxylin and eosin staining (H&E).

Histopathological analysis of the lungs of SARS-CoV-2-infected animals showed interstitial pneumonia, diffuse or not, with bronchoalveolar involvement, oedema of the interstitium and perivascular space, leukocyte infiltration, desquamation of the bronchial epithelium, diffuse interstitial haemorrhage and hyaline membrane deposition (Fig. 4, Table S1). SARS-CoV-2-infected, nontreated animals showed a more marked evolution of the diffuse interstitial pneumonia pattern, bronchitis and oedema scores mainly on Days 3 and 5 p.i. than SARS-CoV-2-infected, serum-treated animals (p value < 0.05).

**Figure 5:**
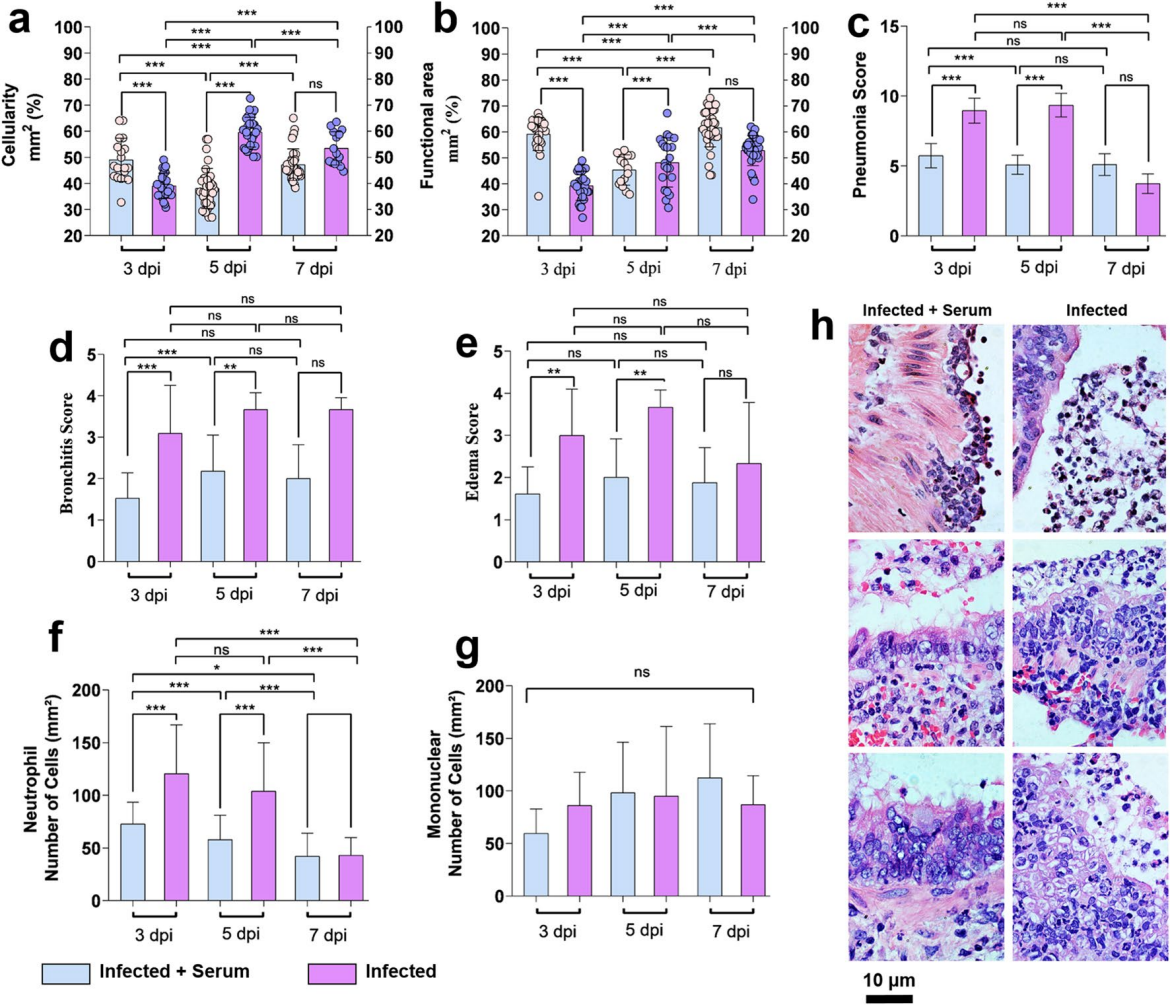
Histopathological changes in hamsters infected and treated with equine anti-SARS-CoV-2 F(ab′)_2_ immunoglobulin. Cellularity (**a**), functional lung area (**b**), pneumonia (**c**), edema (**d**) and bronchitis (**e**) scores. Infiltration kinetics of leukocytes (**f**), neutrophils, and (**g**) mononuclear cells. Evolution of lesions in the bronchial epithelium (**h**). Data are presented as the means ± standard deviations of samples of six animals per group for the scores and five fields/images per slide. The data were analysed statistically with one-way ANOVA, followed by Tukey’s post-test. ***p < 0.0001; ** p < 0.001. Bar 10 µm; haematoxylin and eosin staining (H&E).

In addition, untreated animals showed increased cellularity represented by matrix cells and leukocyte infiltrates and reduced functional pulmonary area represented by the measurement of respiratory areas (intra-alveolar and bronchial area) (p value < 0.05) (Fig. 5).

On Days 3 and 5 p.i., SARS-CoV-2-infected, nontreated animals exhibited marked neutrophilia compared to SARS-CoV-2-infected, serum-treated animals. The number of mononuclear cells increased only at 7 p.i. in SARS-CoV-2-infected, serum-treated animals, although the difference was not significant. Control samples (noninfected animals, with or without treatment) showed the normal histological pattern of the tissue (Fig. 5).

### Detection of antibodies in the hamster COVID model

**Figure 6:**
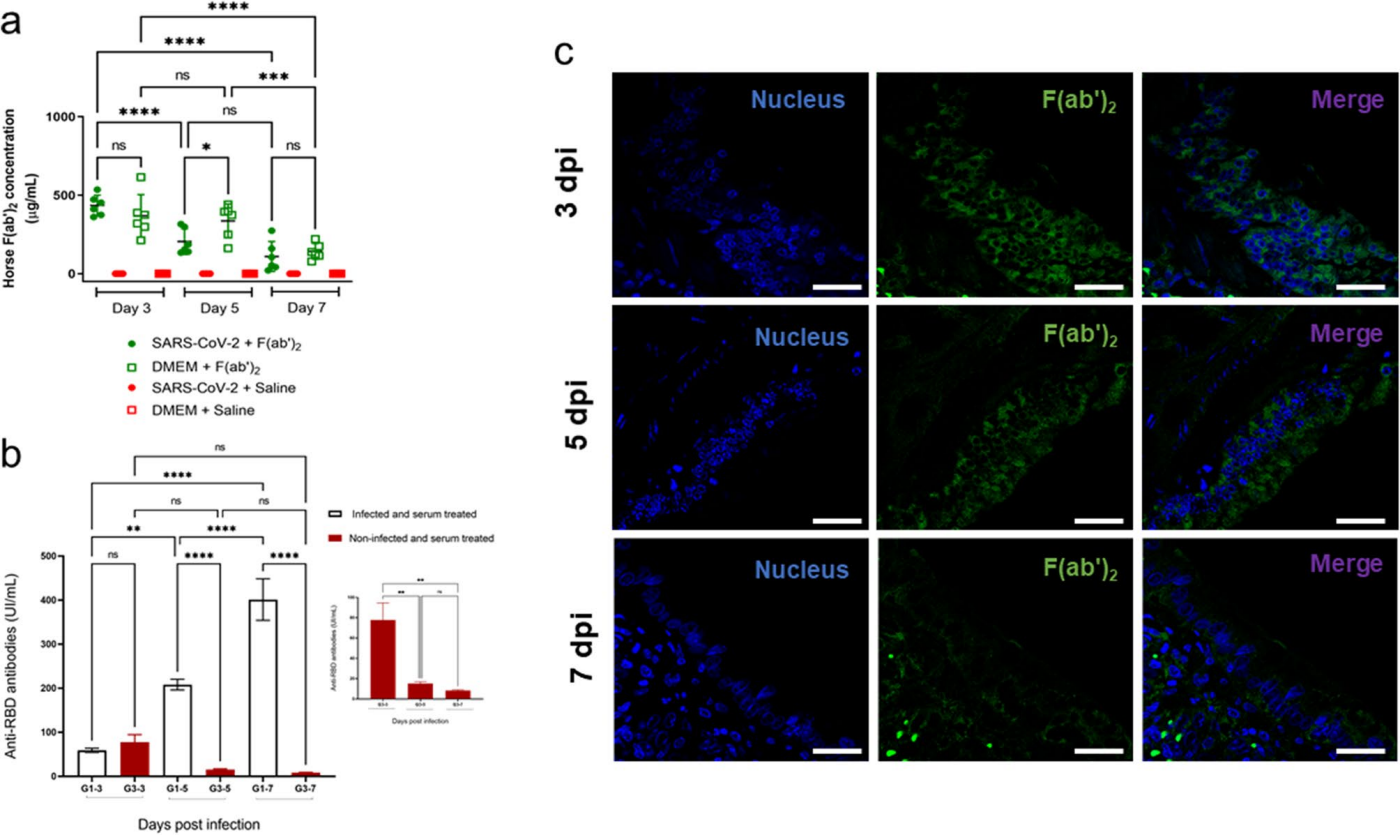
Detection of antibodies in the Golden Syrian hamster COVID model. (**a**) ELISA plates were coated with 100 µL of rabbit anti-horse IgG and then incubated with a previously prepared standard curve consisting of known concentrations of anti-SARS-CoV-2 horse F(ab′)_2_ parallel to hamster serum samples. Horse F(ab′)_2_ in the standard curve or in the samples was then detected by adding peroxidase-conjugated anti-horse IgG followed by the TMB substrate. Absorbance values were measured at 450 nm. The standard curve was linearized by log–log transformation and then subjected to linear regression analysis. The concentration of horse F(ab′)_2_ in hamster serum samples was estimated by interpolating the samples to the standard curve. (**b**) The cPass™ kit for SARS-CoV-2 neutralizing antibody detection was used according to the manufacturer’s instructions for qualitative direct detection of total neutralizing antibodies to SARS-CoV-2 in hamster sera. (**c**) Positive immunofluorescence for F(ab′)_2_ in the lungs of the noninfected group. Thin lung tissue sections from the noninfected group treated with anti-SARS-CoV-2 equine serum were stained with anti-SARS-CoV-2 spike glycoprotein rabbit antibody for one hour and then washed and treated with an Alexa Fluor 647-conjugated anti-rabbit antibody for 30 min. After washing, anti-horse IgG FITC was added, incubated for one hour and washed, and the slides were mounted using Hoechst. The sections were visualized under a confocal microscope (Leica TSC SP8 DSL Hyvolution).

The detection of horse anti-SARS-CoV-2 F(ab′)_2_ in hamster serum samples revealed the presence of horse immunoglobulin fragments, confirming that the intraperitoneally injected anti-SARS-CoV-2 serum reached the blood circulation. The highest concentration was detected at 3 Days p.i. both in infected and noninfected groups and decreased over time in a similar pattern between the groups (Fig. 6A). Moreover, increased amounts of neutralizing antibodies against SARS-CoV-2 in hamster serum were detected on Days 3, 5 and 7 postinfection in animals infected and treated with equine serum. On the other hand, decreased amounts of neutralizing antibodies were detected in noninfected animals treated with equine serum. Nonetheless, no significant differences in the level of neutralizing antibodies were observed on Day 3 p.i. in control or infected animals treated with serum (Fig. 6B). In addition, immunofluorescence staining of the lungs of noninfected animals treated with horse anti-SARS-CoV-2 F(ab′)2 showed that these antibodies were detected on Day 3 p.i., 24 h after antiserum inoculation, but decreased over time (Fig. 6C).

**Figure 7:**
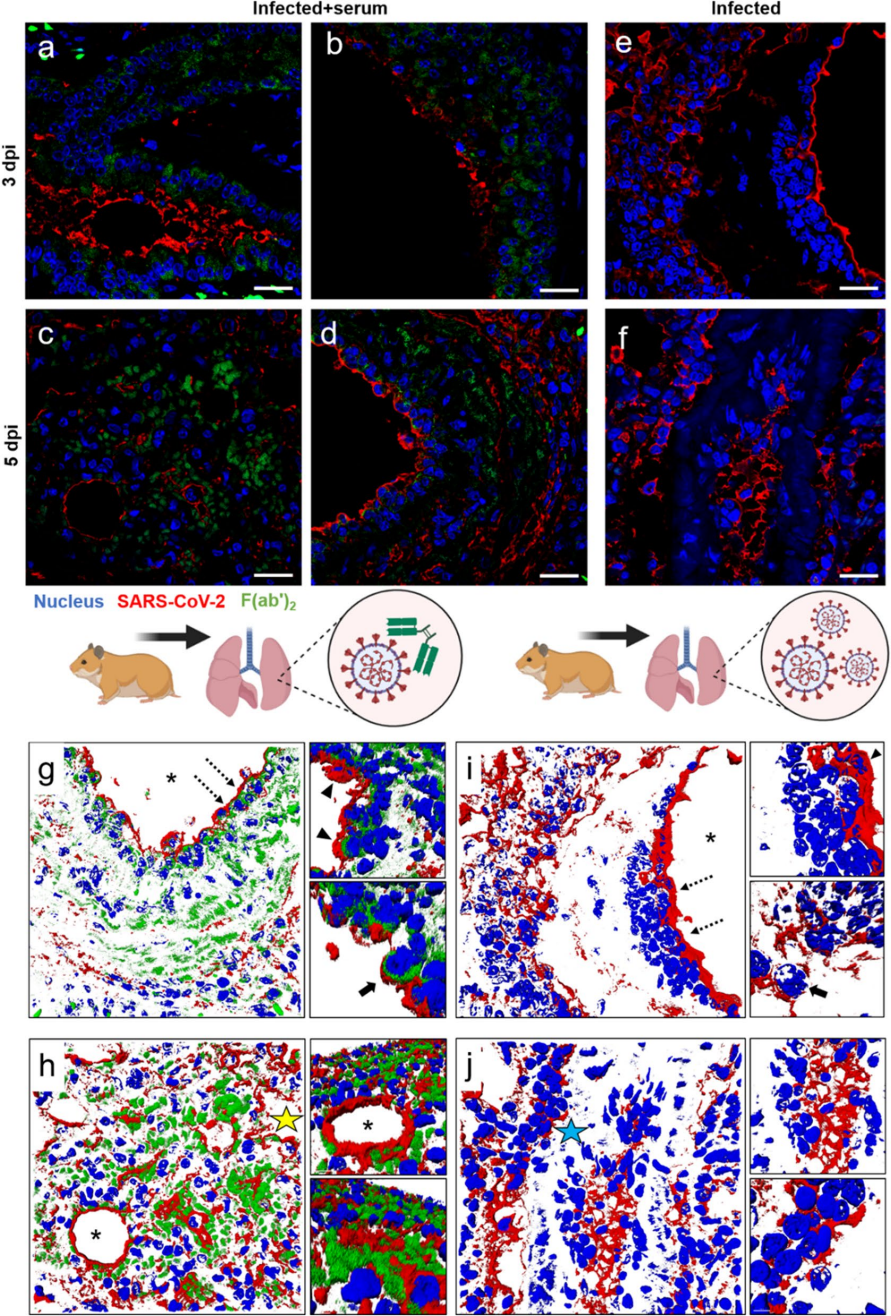
Protective effect of the anti-SARS-CoV-2 antibody on the lungs of the Golden Syrian hamster COVID model. (**a**‒**d**) Positive immunostaining for Spike protein and F(ab′)_2_ in lung tissue from infected animals treated with serum. (**g**‒**h**) 3D analysis showing the detailed interaction of SARS-CoV-2 with F(ab′)_2_. (**e**‒**f**) Distribution of SARS-CoV-2 in experimentally infected lung tissue and (**i**‒**j**) 3D infection kinetics. (*) Lumen of the bronchioles or alveoli; (yellow star) interstice; (blue star) vascular endothelium; (dotted arrows) the area of highest magnification to the right; (arrowhead) regions of viral adhesion and antibodies; (arrow): protective effect of F(ab′)_2_ on a bronchial epithelial cell or just infected cell. Bar: 20 µm.

The kinetics of the infection and the protective effect of the immunotherapy on the lung tissue are shown in Fig. 7. SARS-CoV-2 infected the bronchial alveolar epithelium and vascular endothelium, and it was detected extracellularly in the interstitium or intracellularly in leukocytes and somatic cells. The F(ab′)_2_ fraction is distributed in the same locations as the virus, and the overlap of the F(ab′)_2_-SARS-CoV-2 complex is shown in detail in the 3D analysis of the pulmonary tissue of the infected animals that were treated with serum. The tissue from the saline control group was incubated with the same primary and secondary antibodies, and no specific reaction was observed (Fig. S6—Supplementary Material).

## Discussion

Here, we showed that horses immunized with purified and inactivated SARS-CoV-2 produced plasma with high titres of antibodies as a starting material for the production of anti-SARS-CoV-2 F(ab′)_2_ immunoglobulins in a GMP environment.

The antigen and the final product were characterized using biochemical, immunological and biological approaches. First, the inactivated antigen was evaluated for residual viral activity after irradiation, showing no evidence of active virus and confirming the efficacy of ionizing radiation for inactivation, as previously described for other viruses and SARS-CoV-2^26–28^.

Inactivated viral vaccines are usually obtained by chemical inactivation of viral particles which may cause the loss of some epitopes or impair the antigen potency. The irradiation approach instead is a physical process which tends to preserve the protein content while damages the genetic material when performed at standardized conditions. In this aspect it is expected that the antigen used for the immunization of horses may have even higher quality than the antigen commonly used in inactivated vaccines in terms of preserved immunological properties.

The process used to produce the purified virus was also efficient at reducing the levels of host cell proteins, ensuring product purity, as verified by MS, SDS-PAGE and quantitative ELISA. Additionally, the antigen had no microbial contaminants, and the endotoxins were within acceptable levels for parenteral products.

Finally, the immunological analysis of the antigen revealed that mice immunized with the inactivated virus showed no signs of toxicity and generated high levels of neutralizing antibodies against SARS-CoV-2, indicating the preservation of crucial epitopes of the virus S protein^29^.

The anti-SARS-CoV-2 F(ab′)_2_ immunoglobulin was evaluated using the standard physicochemical and microbiological analysis performed for all 13 immunoglobulin products in the Butantan Institute portfolio. The new product showed no marked differences in the quality analysis and was considered appropriate for the quality control release of anti-SARS-CoV-2 immunoglobulin batches. Particularly for phenol, our anti-SARS-CoV-2 F(ab′)_2_ IgG presented lower concentration per vial than the maximum value specified by the national Pharmacopeia, thus assuring that quantity added is far from the potential toxic limit. Although differences in potency were observed, the product was able to neutralize three SARS-CoV-2 variants in vitro, similar to results obtained for several tests used to establish vaccine efficacy in humans against each new virus variant^30,31^.

Preclinical safety studies performed according to the guidelines of the Brazilian Regulatory Agency using mice and rabbits did not show any toxic effect of the equine anti-SARS-CoV-2 F(ab′)_2_ immunoglobulin (data not shown).

Golden Syrian hamsters were used for the preclinical evaluation of the protective efficacy of anti-SARS-CoV-2 equine serum. An analysis of the viral load in the lung measured using qRT-PCR 3 days after infection showed a significant difference (p < 0.05) between the animals treated with and without the antiserum. Nonetheless, no significant difference in the viral load in trachea and nasal turbinates was observed between groups, as measured using qRT-PCR and by determining the TCID_50_.

Importantly, the challenge studies were conducted using a single antiserum dosage, which was planned to be a lower dose relative to the body weight of a human subject that could be injected. This dosage may explain the lack of a noticeable reduction in viral load, as in vitro studies showed that the serum batches have high potency for neutralization. Nonetheless, anti-SARS-CoV-2 equine antibodies were already detected in the lung 24 h after serum administration (Day 3 p.i.), when nonendogenous anti-SARS-CoV-2 hamster antibodies were present and in which a significant difference in the viral load was determined in the lung. These data reinforce the protective role of the anti-SARS-CoV-2 F(ab′)_2_ immunoglobulins in controlling COVID-19 in the hamster model. On Days 5 and 7 p.i., hamster neutralizing antibodies were present, as along with decreased levels of equine antibodies.

The histopathological analysis showed that equine serum treatment administered 48 h after SARS-CoV-2 infection impaired disease development in hamsters, significantly reducing oedema and neutrophil infiltration into the lung. These data support the potential benefits of this treatment for human patients, who generally suffer from COVID-19-triggered inflammation and the formation of neutrophil extracellular traps^32^. The distribution of the F(ab′)_2_ fraction in the bronchial alveolar epithelium and endothelium, which was observed using immunofluorescence staining, showed the formation of a barrier that seemed to exert a protective effect on the lung tissue. In addition, the sequestration of F(ab′)_2_ in the lung tissue might explain its reduction in hamster serum that was observed using the ELISA.

Although the effects of immunotherapy on the lung tissue have been described in the literature, generally by evaluating the degree of injury and leukocyte kinetics^33–35^, in the present study, we were able to show in situ formation of the F(ab′)_2_-SARS-CoV-2 complex, thus providing new information on the pharmacokinetics of immunotherapy against SARS-CoV-2.

In clinical practice, antibodies originating from horses immunized with animal venoms, bacterial toxins and rabies virus have been used since the introduction of the first diphtheria and tetanus antitoxins in the nineteenth century. Formerly, the use of equine-derived immunoglobulins produced considerable problems related to anaphylactic and non-anaphylactic early reactions and serum sickness, likely related to the substantial volumes of product injected. Purification steps have generated products of much higher quality, and adverse reactions have decreased in frequency and severity. Thus, antivenoms remain the cornerstone in the treatment of snakebite envenomation worldwide, while antitoxins and equine anti-rabies immunoglobulin are still widely used, especially in low- and middle-income countries. Equine antibodies can also be administered prophylactically after exposure to the organism, as in postexposure rabies prophylaxis, in immunodeficient or immunosuppressed subjects or in areas where no vaccines are available. A further possible use of equine antibody therapy, in this case for COVID-19 infection, is when patients become infected while still protected by the antibodies. In this case, antibodies would functions as surrogate vaccines, as observed for IgG antibodies against flu, which protect the lung from disease but do not protect the nose from infection in mice^36^.

A phase I/II clinical trial aiming to assess the safety, pharmacokinetics and efficacy of our equine anti-SARS-CoV-2 F(ab′)_2_ IgG in patients with an early stage of COVID-19 and increased risk of developing severe illness (NCT0449484) has been recently approved by the Brazilian regulatory authority. We expect to determine its benefit as a therapeutic tool in reducing the morbidity and mortality caused by COVID-19. The large-scale production of anti-SARS-CoV-2 serum may be more affordable than convalescent plasma or monoclonal antibodies, increasing the availability and accessibility of an effective alternative to treat patients in low- and middle-income countries.

## Conclusions

Here, we describe the development of an anti-SARS-CoV-2 equine F(ab′)_2_ immunoglobulin using a newly developed SARS-CoV-2 viral antigen that was purified and inactivated by radiation. The obtained product recognized and neutralized the original virus and variants in vitro. In animal challenge tests, colocalization of the virus and the equine antibody in the lung tissue and a reduction in the severity of the disease were observed.

## Supplementary Information

Supplementary Information.

## Data Availability

The original contributions presented in the study are included in the article/Supplementary Material, further inquiries can be directed to the corresponding authors.
