# Supplementary material for: Anti-SARS-CoV-2 equine F (Ab′)2 immunoglobulin as a possible therapy for COVID-19
Source: Sci Rep. 2022 Mar 10;12:3890. doi: 10.1038/s41598-022-07793-1 (PMC8913704; doi:10.1038/s41598-022-07793-1)
Supplement: Supplementary file 1 — Supplementary Information. [file 41598_2022_7793_MOESM1_ESM.docx]

**Anti-SARS-CoV-2 Equine F (Ab’)_2_ Immunoglobulin as a Possible Therapy for COVID-19**

Viviane Fongaro Botosso^1¶^, Soraia Attie Calil Jorge^2¶^, Renato Mancini Astray^3¶^, Ana Marcia de Sá Guimarães^4¶^, Monica Beatriz Mathor^5¶^, Patrícia dos Santos de Carneiro^6¶^, Edison Luiz Durigon^4^, Dimas Covas^7^, Danielle Bruna Leal de Oliveira^4,1^, Ricardo das Neves Oliveira^8^, Durvanei Augusto Maria^9^, Silas Fernandes Eto^9^, Neuza Maria Frazatti Gallina^10^, Giselle Pidde^11^, Carla Cristina Squaiella-Baptistão^11^, Dilza Trevisan Silva^12^, Isadora Maria Villas-Boas^11^, Dayanne Carla Fernandes^11^, Aline Vivian Vatti Auada^8^, Alexandre Campos Banari^4^, Antônio Francisco de Souza Filho^4^, Camila Bianconi^8^, Carla Lilian de Agostini Utescher^1^, Denise Cristina André Oliveira^1^, Douglas Oscar Ceolin Mariano^8^, Flávia Ferreira Barbosa^3^, Giuliana Rondon^9^, Josana Kapronezai^1^, Juliana Galvão da Silva^6^, Mauricio Barbugiani Goldfeder^9^, Priscila Comone^8^, Regis Edgar Castilho Junior^9^, Taiana Tainá Silva Pereira^4^, Fan Hui Wen^8^**^*^**, Denise V. Tambourgi^11^**^*^**, Ana Marisa Chudzinski-Tavassi^9,12^**^*^**

**Supplementary Information**

**Supplementary Tables**

**Table S1.**

Histopathology scores of *Mesocricetus auratus* infected and submitted to serum therapy with equine Anti-SARS-CoV-2 F(ab´)_2_ immunoglobulins.

| Histopathological findings |  | Infected + Serum |  |  |  | Infected |  |
| --- | --- | --- | --- | --- | --- | --- | --- |
|  | **3 dpi** | **5 dpi** | **7 dpi** |  | **3 dpi** | **5 dpi** | **7 dpi** |
| Edema | 5,83 | 5,6 | 4,17 |  | 10,33 | 10 | 3,67 |
| Consolidation (Pneumonia and bronchopneumonia) | 6,5 | 5,5 | 6 |  | 10,17 | 10,17 | 4 |
| Alveolar hemorrhage | 5,33 | 3,4 | 4,8 |  | 9,17 | 7,83 | 3,33 |
| Alveolar hyaline membrane | 3,83 | 5,33 | 6,33 |  | 8,83 | 8,83 | 3,33 |
| Inflammation | 7,17 | 5,2 | 4,8 |  | 9,67 | 10,5 | 3 |
| Necrosis and septal destruction | 5,33 | 5,4 | 5,2 |  | 8 | 9,83 | 3,67 |
| Hyperplasia | 5,17 | 5,2 | 5,5 |  | 8,17 | 9 | 4,33 |
| Thickening of the alveolar wall | 5,33 | 5,2 | 5 |  | 9 | 8,33 | 4,33 |
| Deposition of extracellular matrix | 4,17 | 5,6 | 5,33 |  | 8,33 | 9,33 | 5 |
| Neutrophilia | 6,67 | 4,4 | 3,8 |  | 7,83 | 9,67 | 2,67 |
| Pulmonary fibrosis | - | - | 3 |  |  | 7,67 | 2 |
| Average | 5,73 | 5,08 | 4,90 |  | 8,95 | 9,20 | 3,58 |
| Standard deviation | 0,87 | 0,68 | 0,96 |  | 0,89 | 0,95 | 0,84 |

Score metric per group 1-3 (mild pneumonia); 4-6 (moderate) and 7-9 (Severe).

**Table S2.**

Animal weight and laboratory parameters of horses submitted to SARS-CoV-2 inactivated virus immunization and plasma collection.

| **Parameters** | **Prior to 1^st^ dose** | **Prior to 4^th^ dose** | **Prior to 1^st^ collection** | **Prior to 2^nd^ collection** | **Prior to 3^rd^ collection** | **1 week after 3^rd^ collection** | **Normal range** |
| --- | --- | --- | --- | --- | --- | --- | --- |
| **Weight** (mean ± SD) | 468.45 ± 32.10 | 480.20 ± 35.58 | 469.30 ± 32.40 | 463.80 ± 31.55 | 478.70 ± 26.3 | 469.8 ± 26.45 | **400-500** |
| **Whole blood cells** (x10^6^ µL) | 8.04 ± 1.24 | 7.55 ± 0.82 | 8.87 ± 1.47 | 9.12 ± 1.24 | 9.02 ± 0.90 | 8.46 ± 1.18 | **6.5-12.5** |
| **Ht** (%) | 37.70 ± 5.25 | 34.20 ± 3.08 | 38.90 ± 5.59 | 41.70 ± 5.52 | 41.80 ± 4.80 | 37.30 ± 4.55 | **32-52** |
| **Leucocytes** ( /µL) | 9.35 ± 1.08 | 10.02 ± 1.00 | 9.35 ± 1.15 | 11.27 ± 1.80 | 11.96 ± 1.75 | 9.79 ± 1.14 | **5.5-12.5** |
| **Platelets** (x10^3^ µL) | 197.80 ± 40.88 | 207.20 ± 59.62 | 180.92 ± 101.84 | 211.20 ± 52.57 | 249.40 ± 26.72 | 225.80 ± 47.14 | **100-350** |
| **BUN** (g/dL) | 38.21 ± 2.22 | 27.66 ± 5.02 | 38.33 ± 4.66 | 28.06 ± 3.87 | 32.98 ± 5.79 | 29.95 ± 5.58 | **20.00-40.00** |
| **Creatinine** (mg/dL) | 1.63 ± 0.21 | 1.64 ± 0.23 | 1.48 ± 0.15 | 1.55 ± 0.12 | 1.53 ± 0.15 | 1.54 ± 0.15 | **1.20-2.00** |
| **Total protein** (g/dL) | 6.99 ± 0.13 | 7.52 ± 0.39 | 6.66 ± 1.16 | 6.69 ± 0.37 | 5.45 ± 0.62 | 6.91 ± 0.24 | **5.2-7.9** |
| **Globulin** (g/dL) | 3.04 ± 0.37 | 3.78 ± 0.46 | 3.05 ± 0.48 | 3.09 ± 0.38 | 2.43 ± 0.48 | 3.35 ± 0.30 | **2.6-4.0** |
| **Albumin** (g/dL) | 3.95 ± 0.26 | 3.73 ± 0.15 | 3.91 ± 0.24 | 2.66 ± 0.20 | 3.01 ± 0.24 | 3.57 ± 0.17 | **2.6-3.7** |
| **AST** (U/L) | 342.96 ± 61.86 | 246.60 ± 60.20 | 420.95 ± 155.47 | 214.46 ± 35.15 | 233.89 ± 40.90 | 282.38 ± 30.11 | **< 230** |
| **ALT** (U/L) | 9.63 ± 2.85 | 8.22 ± 2.32 | 7.75 ± 2.18 | 4.74 ± 1.18 | 6.46 ± 2.14 | 7.66 ± 1.47 | **< 175** |
| **Total bilirubin** (U/L) | 0.75 ± 0.21 | 0.96 ± 0.28 | 1.47 ± 0.34 | 1.12 ± 0.34 | 1.47 ± 0.34 | 1.07 ± 0.23 | **0-2.0** |
| **Indirect bilirubin** (U/L) | 0.55 ± 0.19 | 0.72 ± 0.25 | 1.09 ± 0.31 | 0.83 ± 0.33 | 1.16 ± 0.34 | 0.70 ± 0.19 | **0-1.6** |
| **K^+^** (mmol/L) | 4.13 ± 0.29 | 3.92 ± 0.32 | 3.67 ± 0.70 | 4.15 ± 0.49 | 4.20 ± 0.41 | 3.59 ± 0.41 | **2.40-4.70** |
| **Na^+^** (mmol/L) | 131.80 ± 2.10 | 130.40 ± 0.52 | 135.50 ± 4.31 | 137.40 ± 5.21 | 132.60 ± 0.97 | 131.20 ± 1.40 | **132-146** |
| **Ca^++^** (mmol/L) | 1.47 ± 0.08 | 1.46 ± 0.03 | 1.44 ± 0.03 | 1.47 ± 0.05 | 1.42 ± 0.05 | 1.44 ± 0.03 | **1.40-1.79** |
| **CK** (U/L) | 370.67 ± 123.00 | 299.14 ± 141.40 | 348.58 ± 82.55 | 254.34 ± 73.08 | 200.98 ± 63.29 | 246.57 5± 9.94 | **< 120** |
| **DHL** (U/L) | 755.24 ± 153.25 | 902.59 ± 68.33 | 728.85 ± 77.13 | 641.25 ± 106.72 | 556.47 ± 97.19 | 675.23 ± 124.74 | **< 570** |
| **LDL** (mg/dL) | 40.78 ± 9.17 | 28.58 ± 4.18 | 35.23 ± 7.96 | 26.80 ± 7.01 | 13.88 ± 4.25 | 17.10 ± 5.53 | **25.7-59.7** |
| **HDL** (mg/dL) | 63.57 ± 2.61 | 71.00 ± 5.23 | 62.51 ± 2.65 | 46.30 ± 4.76 | 55.60 ± 10.02 | 72.30 ± 9.98 | **60.4-104.9** |
| **Glucose** (mg/dL) | 97.88 ± 14.39 | 82.00 ± 2.70 | 111.15 ± 15.02 | 109.58 ± 16.67 | 95.46 ± 19.99 | 91.37 ± 8.70 | **75-115** |
| **TP** (sec) | 11.48 ± 0.60 | 10.97 ± 0.71 | 9.82 ± 0.41 | 10.87 ± 0.52 | 10.96 ± 0.98 | 10.19 ± 0.51 | 9-12 |
| **ATTP** (sec) | 40.86 ± 2.69 | 38.47 ± 4.25 | 39.51 ± 3.45 | 36.70 ± 4.13 | 41.37 ± 4.08 | 41.10 ± 3.53 | 27-45 |

**Table S3.**

Collection parameters of the three pools of anti-SARS-CoV-2 hyperimmune plasma

| Pool of plasma | Total volume collected (mL) *per* animal | Time of collection* (min) | Volume of plasma (mL) *per* kg of animal weight* | Rate of collection* (mL/min) |
| --- | --- | --- | --- | --- |
| 1 | 15,082 | 257 ± 30.0 | 30.0 ± 1.8 | 54.5 ± 4.5 |
| 2 | 14,923 | 252.4 ± 27.6 | 32.5 ± 1.8 | 59.1 ± 6.4 |
| 3 | 12,710 | 230.0 ± 19.9 | 26.6 ± 4.2 | 55.1 ± 7.2 |

* Mean ± SD

**Table S4**

SARS-CoV-2 load in lungs, nasal turbinates (NT), and trachea of Golden Syrian Hamsters measured at days 3. 5 and 7 p.i. Group 1 – SARS-CoV-2 infected animals and treated with anti-SARS-CoV-2 equine serum at day 2 p.i.; Group 2 – SARS-CoV-2 infected animals and non-treated with anti-SARS-CoV-2 equine serum; Group 3 – non-infected animals and treated with anti-SARS-CoV-2 equine serum at day 2 p.i.; group 4 – non-infected animals and non-treated with anti-SARS-CoV-2 equine serum. Viral load is expressed in log TCID50 (Tissue Culture Infective Dose 50%)/gram of tissue and in number of SARS-CoV-2 RNA copies per number of RNA β actin copies as measured through RT-qPCR per gram of tissue. ND – not detected.

| log TCID 50/g tissue | | | | | | | | | | | | | | |
| --- | --- | --- | --- | --- | --- | --- | --- | --- | --- | --- | --- | --- | --- | --- |
| Lung | | | | | | | | | | | | | | |
| Group 1 | | |  | Group 2 | | |  | Group 3 | | |  | Group 4 | | |
| day 3 | day 5 | day 7 |  | day 3 | day 5 | day 7 |  | day 3 | day 5 | day 7 |  | day 3 | day 5 | day 7 |
| 10,9 | 8,3 | 0,0 |  | 12,1 | 7,5 | 0,0 |  | ND | ND | ND |  | ND | ND | ND |
| 12,9 | 8,1 | 0,0 |  | 9,0 | 7,2 | 0,0 |  | ND | ND | ND |  | ND | ND | ND |
| 8,5 | 7,3 | 4,5 |  | 13,2 | 8,1 | 0,0 |  | ND | ND | ND |  | ND | ND | ND |
| 9,9 | 6,7 | 0,0 |  | 12,7 | 7,0 | 0,0 |  | ND | ND | ND |  | ND | ND | ND |
| 9,6 | 7,0 | 0,0 |  | 12,4 | 8,0 | 0,0 |  | ND | ND | ND |  | ND | ND | ND |
| 9,4 | 6,9 | 0,0 |  | 14,8 | 5,7 | 0,0 |  | ND | ND | ND |  | ND | ND | ND |
|  |  |  |  |  |  |  |  |  |  |  |  |  |  |  |
| Nasal Turbinate | | | | | | | | | | | | | | |
| Group 1 | | |  | Group 2 | | |  | Group 3 | | |  | Group 4 | | |
| day 3 | day 5 | day 7 |  | day 3 | day 5 | day 7 |  | day 3 | day 5 | day 7 |  | day 3 | day 5 | day 7 |
| 12,1 | 5,3 | 0,0 |  | 7,3 | 4,8 | 0,0 |  | ND | ND | ND |  | ND | ND | ND |
| 7,2 | 2,6 | 5,2 |  | 7,7 | 3,5 | 0,0 |  | ND | ND | ND |  | ND | ND | ND |
| 8,1 | 3,2 | 1,4 |  | 8,6 | 6,1 | 0,0 |  | ND | ND | ND |  | ND | ND | ND |
| 8,1 | 5,6 | 0,0 |  | 8,6 | 5,0 | 0,0 |  | ND | ND | ND |  | ND | ND | ND |
| 6,6 | 2,8 | 0,0 |  | 6,3 | 4,6 | 0,0 |  | ND | ND | ND |  | ND | ND | ND |
| 7,5 | 6,0 | 0,0 |  | 7,1 | 0,0 | 0,0 |  | ND | ND | ND |  | ND | ND | ND |
|  |  |  |  |  |  |  |  |  |  |  |  |  |  |  |
| Trachea | | | | | | | | | | | | | | |
| Group 1 | | |  | Group 2 | | |  | Group 3 | | |  | Group 4 | | |
| day 3 | day 5 | day 7 |  | day 3 | day 5 | day 7 |  | day 3 | day 5 | day 7 |  | day 3 | day 5 | day 7 |
| 4,7 | 3,9 | 0,0 |  | 3,9 | 3,0 | 0,0 |  | ND | ND | ND |  | ND | ND | ND |
| 6,7 | 6,9 | 0,0 |  | 6,9 | 2,8 | 0,0 |  | ND | ND | ND |  | ND | ND | ND |
| 6,3 | 6,8 | 0,0 |  | 6,8 | 2,6 | 0,0 |  | ND | ND | ND |  | ND | ND | ND |
| 5,4 | 6,8 | 0,0 |  | 6,8 | 0,0 | 0,0 |  | ND | ND | ND |  | ND | ND | ND |
| 2,4 | 5,7 | 0,0 |  | 5,7 | 3,0 | 0,0 |  | ND | ND | ND |  | ND | ND | ND |
| 5,8 | 5,2 | 0,0 |  | 5,2 | 2,9 | 0,0 |  | ND | ND | ND |  | ND | ND | ND |

| log Viral RNA copies /βactina RNA copies/g | | | | | | | | | | | | | | |
| --- | --- | --- | --- | --- | --- | --- | --- | --- | --- | --- | --- | --- | --- | --- |
| Lung | | | | | | | | | | | | | | |
| Group 1 | | |  | Group 2 | | |  | Group 3 | | |  | Group 4 | | |
| day 3 | day 5 | day 7 |  | day 3 | day 5 | day 7 |  | day 3 | day 5 | day 7 |  | day 3 | day 5 | day 7 |
| 2,5 | 1,6 | -0,6 |  | 3,06 | 2,39 | 0,0 |  | ND | ND | ND |  | ND | ND | ND |
| 2,5 | 1,1 | -0,3 |  | 2,46 | 2,01 | -0,7 |  | ND | ND | ND |  | ND | ND | ND |
| 2,1 | 2,1 | 1,0 |  | 3,08 | 2,31 | -0,1 |  | ND | ND | ND |  | ND | ND | ND |
| 1,7 | 1,7 | 0,0 |  | 2,17 | 1,24 | -2,2 |  | ND | ND | ND |  | ND | ND | ND |
| 2,1 | 1,6 | -0,8 |  | 2,92 | 1,79 | -1,8 |  | ND | ND | ND |  | ND | ND | ND |
| 1,2 | 1,2 | -0,4 |  | 3,02 | 1,28 | -0,3 |  | ND | ND | ND |  | ND | ND | ND |
|  |  |  |  |  |  |  |  |  |  |  |  |  |  |  |
| Nasal Turbinate | | | | | | | | | | | | | | |
| Group 1 | | |  | Group 2 | | |  | Group 3 | | |  | Group 4 | | |
| day 3 | day 5 | day 7 |  | day 3 | day 5 | day 7 |  | day 3 | day 5 | day 7 |  | day 3 | day 5 | day 7 |
| 3,21 | 2,50 | 0,63 |  | 2,97 | 1,76 | -0,03 |  | ND | ND | ND |  | ND | ND | ND |
| 2,90 | 0,22 | 0,03 |  | 3,03 | 2,50 | -0,65 |  | ND | ND | ND |  | ND | ND | ND |
| 2,11 | 2,77 | 0,04 |  | 3,30 | 2,27 | -0,10 |  | ND | ND | ND |  | ND | ND | ND |
| 3,29 | 1,71 | 0,19 |  | 3,17 | 2,02 | -2,20 |  | ND | ND | ND |  | ND | ND | ND |
| 2,71 | 0,77 | 0,52 |  | 2,33 | 1,78 | -1,84 |  | ND | ND | ND |  | ND | ND | ND |
| 2,60 | 1,84 | 0,22 |  | 3,01 | 1,62 | -0,33 |  | ND | ND | ND |  | ND | ND | ND |
|  |  |  |  |  |  |  |  |  |  |  |  |  |  |  |
| Trachea | | | | | | | | | | | | | | |
| Group 1 | | |  | Group 2 | | |  | Group 3 | | |  | Group 4 | | |
| day 3 | day 5 | day 7 |  | day 3 | day 5 | day 7 |  | day 3 | day 5 | day 7 |  | day 3 | day 5 | day 7 |
| 0,00 | 2,61 | -2,10 |  | 1,89 | 1,71 | -0,91 |  | ND | ND | ND |  | ND | ND | ND |
| 3,08 | 2,07 | -1,49 |  | 2,86 | 0,80 | -2,35 |  | ND | ND | ND |  | ND | ND | ND |
| 2,08 | 1,13 | 1,50 |  | 2,82 | 0,35 | 0,12 |  | ND | ND | ND |  | ND | ND | ND |
| 2,38 | 0,69 | -0,21 |  | 0,81 | 1,51 | -1,90 |  | ND | ND | ND |  | ND | ND | ND |
| -0,46 | -0,95 | -1,08 |  | -0,57 | 1,51 | 0,00 |  | ND | ND | ND |  | ND | ND | ND |
| 1,93 | 1,73 | 0,00 |  | 0,05 | -0,61 | 0,00 |  | ND | ND | ND |  | ND | ND | ND |

**Supplementary figures**

**
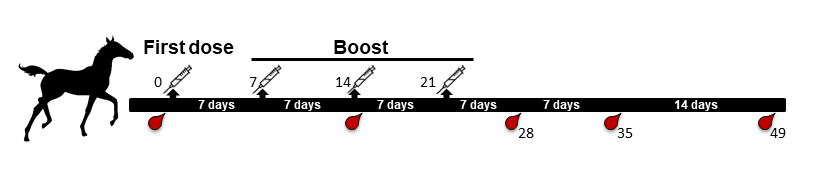
Figure S1.** Timeline for immunizing (via subcutaneous) horses with inactivated and purified SARS-CoV-2. Red drops indicate blood collections, while syringes indicate antigen inoculations.


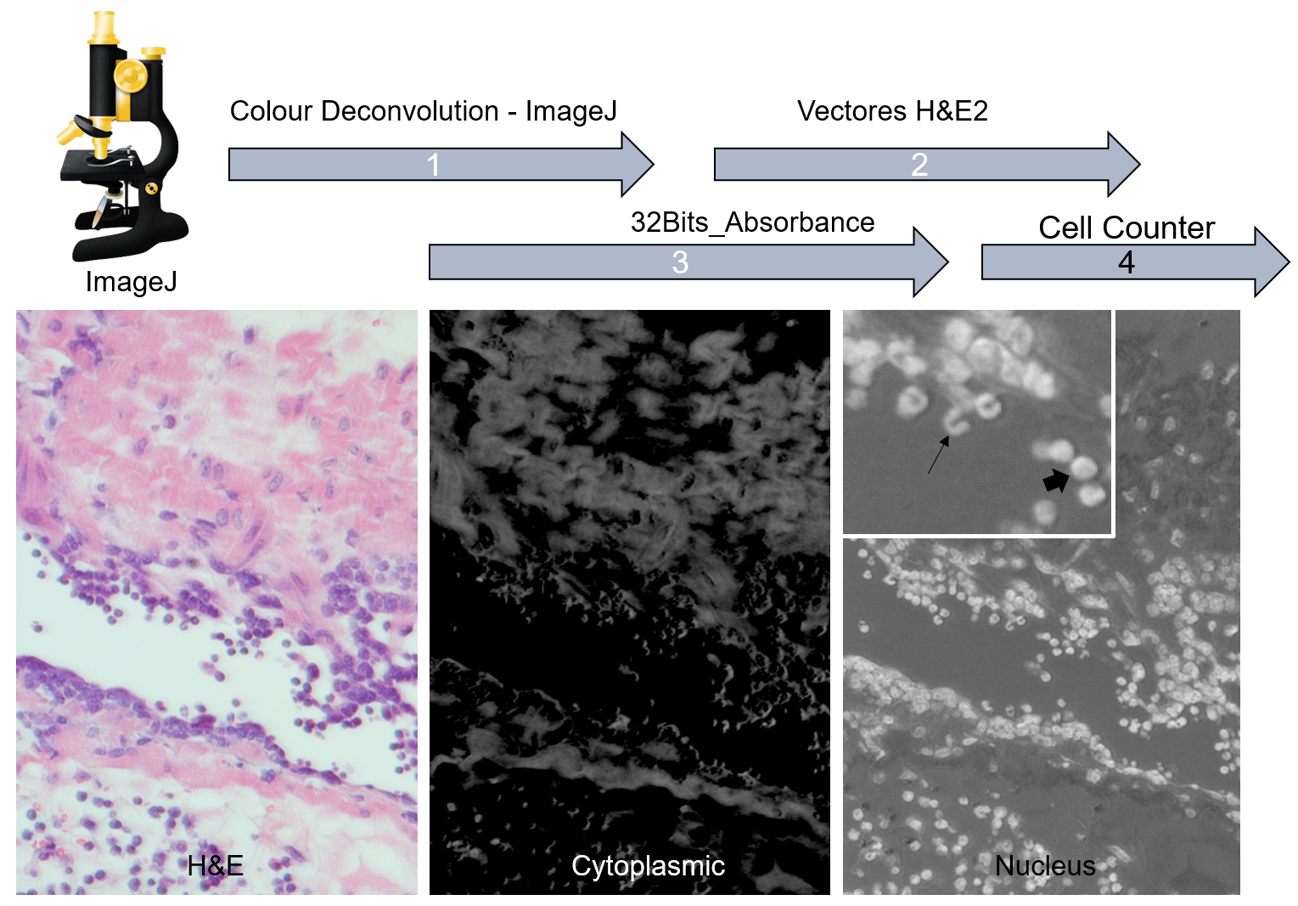


**Figure S2.** Methodology for counting leukocytes in the lung tissue of Golden Syrian hamsters infected with SARS-CoV-2. (1) separation of nuclei and cytoplasm by deconvolution; (2) selection of H & E2 vectors; exposure and transformation of the image into absorption in 32 bits and (4) counting of cells by Cell Counter. (→) segmented nucleus (⇨) mononuclear.

**
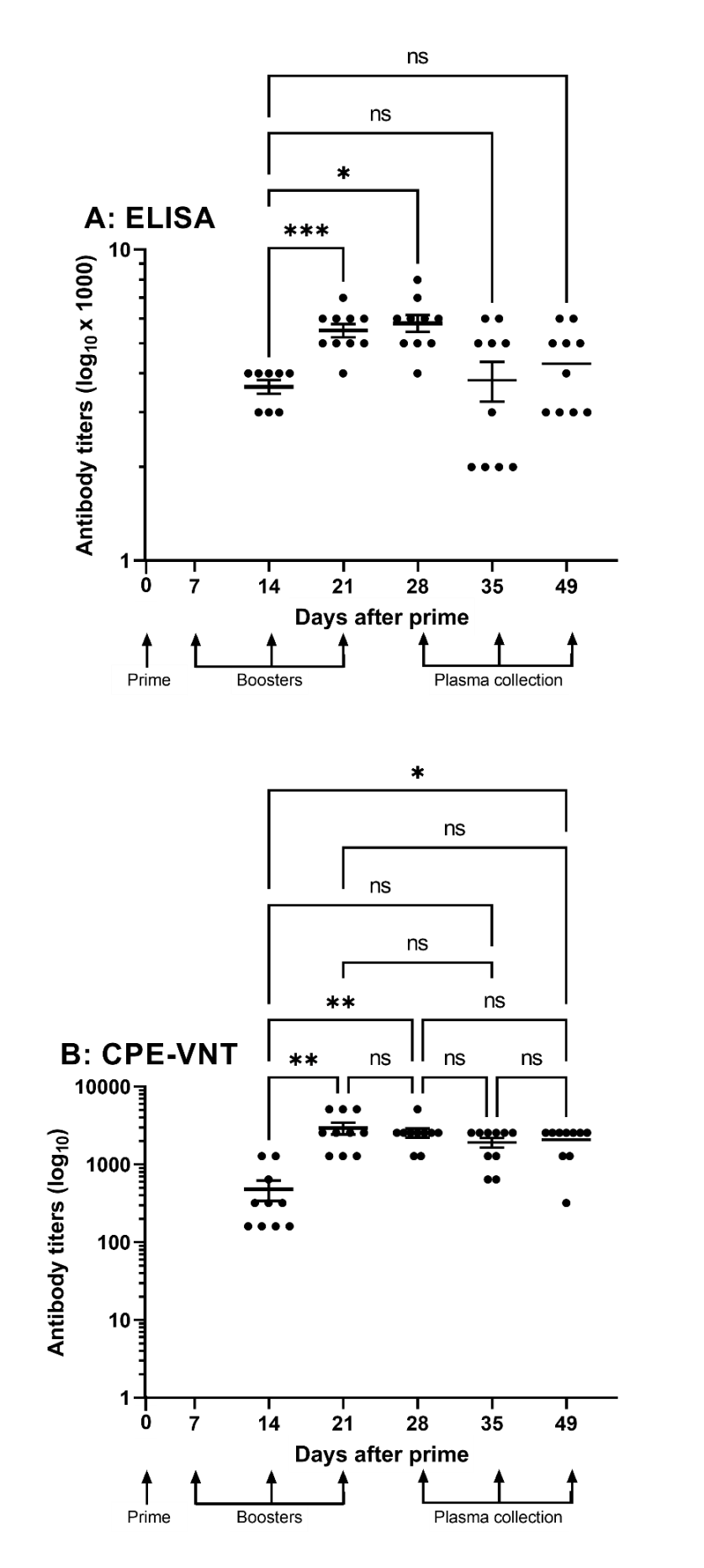
**

**Figure S3.** Titers of the anti-SARS-CoV-2 horse hyperimmune plasma. [A] ELISA plates were coated with 100 µL of purified and inactivated SARS-CoV-2 (2 x 10^3^ virus/mL) and incubated with increasing dilutions of the pre-immune or experimental sera, obtained from the horses hyperimmunized with the inactivated SARS-CoV-2. Then the plates were incubated with anti-horse HRPO-conjugated IgG. The reaction was performed with the addition of TMB substrate, and spectrophotometric readings were taken at λ 450 nm. The titer was established as the highest serum dilution in which the measured absorbance was twice as high as that determined for pre-immune serum (control group). [B] Sera from horse immunized with purified and inactivated SARS CoV-2 antigen were also tested in the CPE-VNT. The neutralization titer was defined by the inverse of the highest dilution of serum that blocks the viral replication. Statistical analyses were performed using *t*-student followed by Mann Whitney test (*p≤ 0.05).

**A**


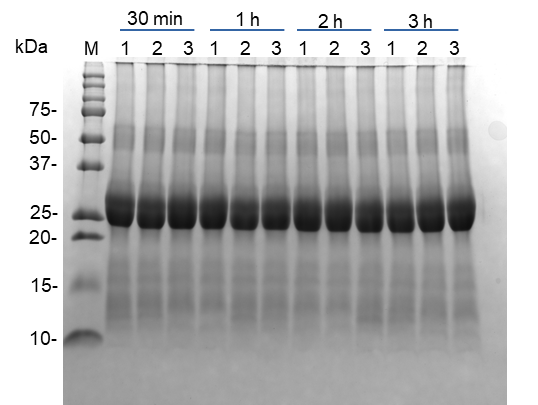

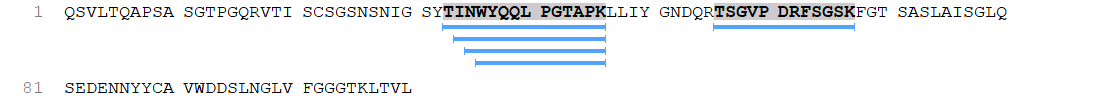


**>QKY75967.1 anti-SARS-CoV-2-spike protein immunoglobulin light chain variable region, partial [*Homo sapiens*]**

**B**

**Figure S4.** Protein profile of anti-SARS-CoV-2 horse hyperimmune serum and identification of specific anti-spike immunoglobulin through LC–MS/MS analysis. [A] SDS–PAGE protein profile: 1, 2 and 3 correspond to samples of batches 0001, 0002 and 0003, respectively. Incubation times at 37 °C are shown above the gel, and the molecular mass markers are indicated on the left side. [B] Example of mass spectrometry protein identification of specific anti-spike immunoglobulins. The same sequence was identified in all analysed batches.

**
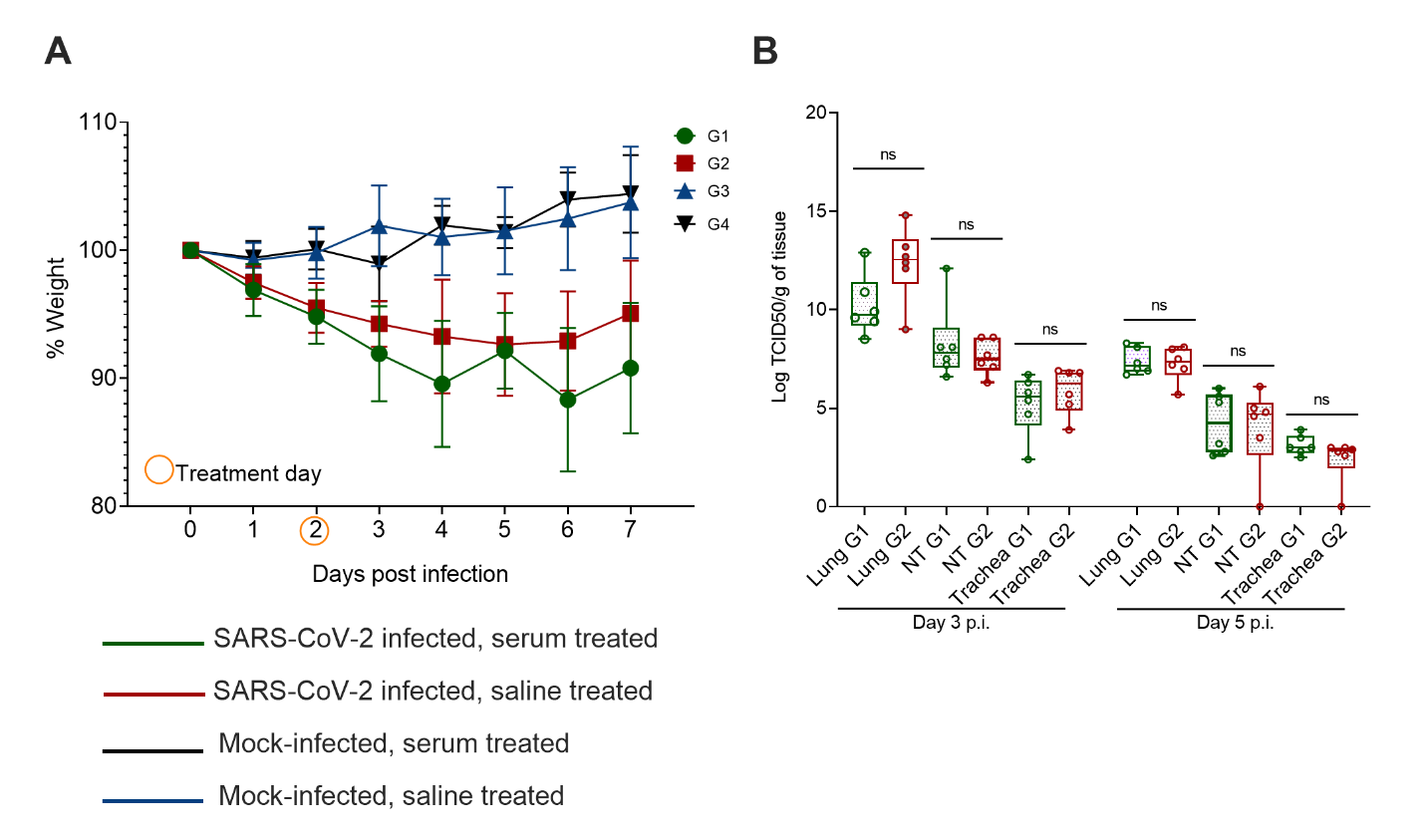
**

**Figure S5.** SARS-CoV-2 challenge test in Golden Syrian Hamsters. [A] Weight changes observed in SARS-CoV-2 infected or non-infected animals, that were submitted or not to the serum treatment. [B] Viral load expressed in 50% Tissue Culture Infective Dose/gram of tissue. Green: G1 animals (n=6 per subgroup), *i.e*., serum-treated, SARS-CoV-2 infected animals; Red: G2 animals (n=6 per subgroup), *i.e*., non-treated, SARS-CoV-2 infected animals. Black: G3 animals (n=6 per subgroup), *i.e*., non-infected, serum treated animals. Blue: G4 animals (n=6 per subgroup), *i.e*., non-infected, non-treated animals. p.i.= post infection; n.s.: not significant (p value > 0.05).


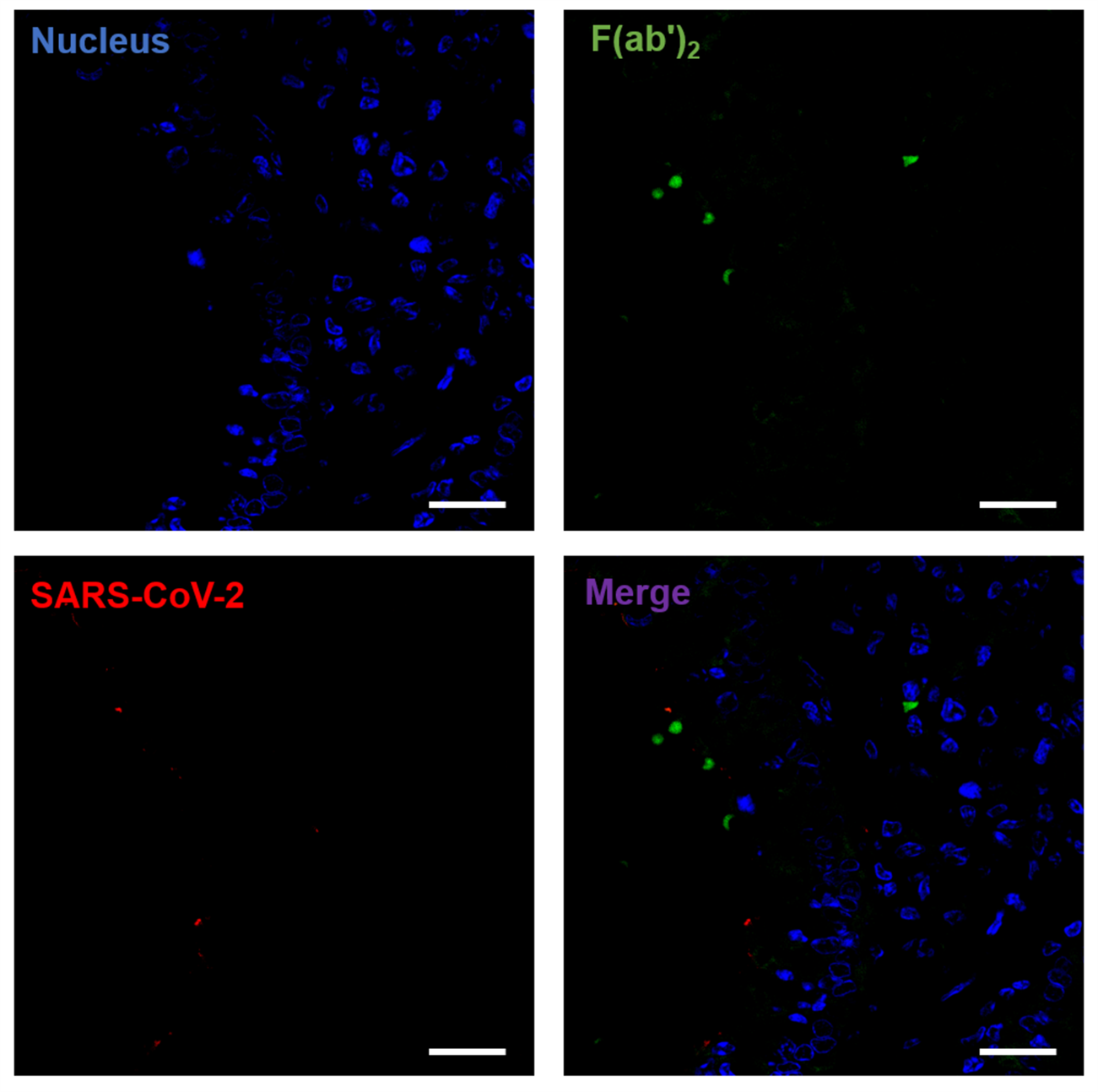


**Figure S6.** Immunofluorescence analysis of lung of non-infected, non-treated hamsters (control). Lung tissue thin sections from hamsters non-infected and non-treated with the equine anti-SARS-CoV-2 (Fab´)_2_ immunoglobulin end-product were treated with anti-SARS-CoV-2 spike glycoprotein rabbit antibody followed by anti-rabbit antibody Alexa Fluor 647. After washing, anti-horse IgG FITC was added, the thin sections were washed and the slides were mounted using Hoechst and ProLong™ Glass Antifade Mountant. The slides were read under a confocal microscope. Bar: 20 µm.


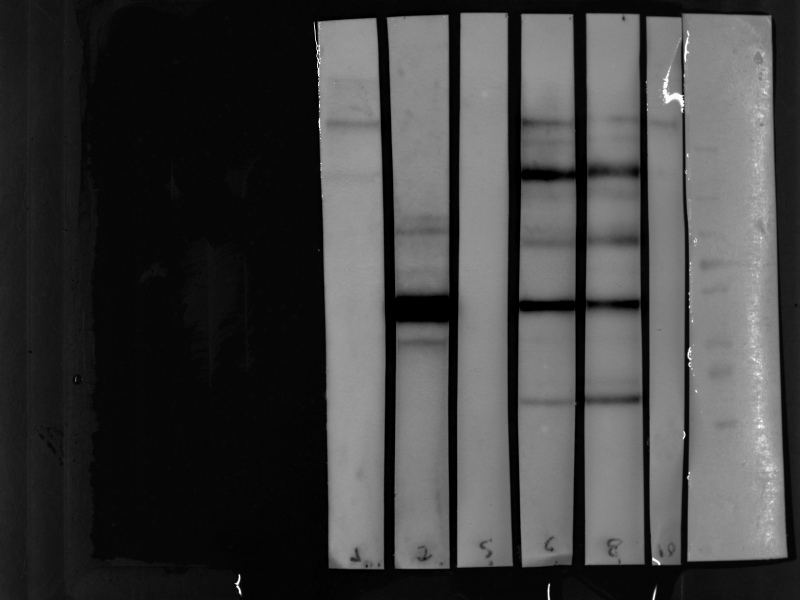

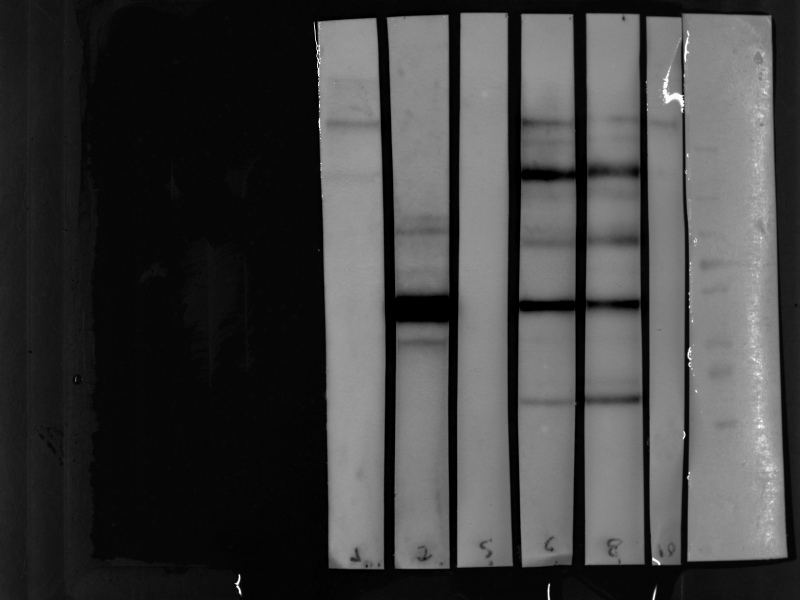


**4 5 6**

250

130

100

70

50

35

25

15

10


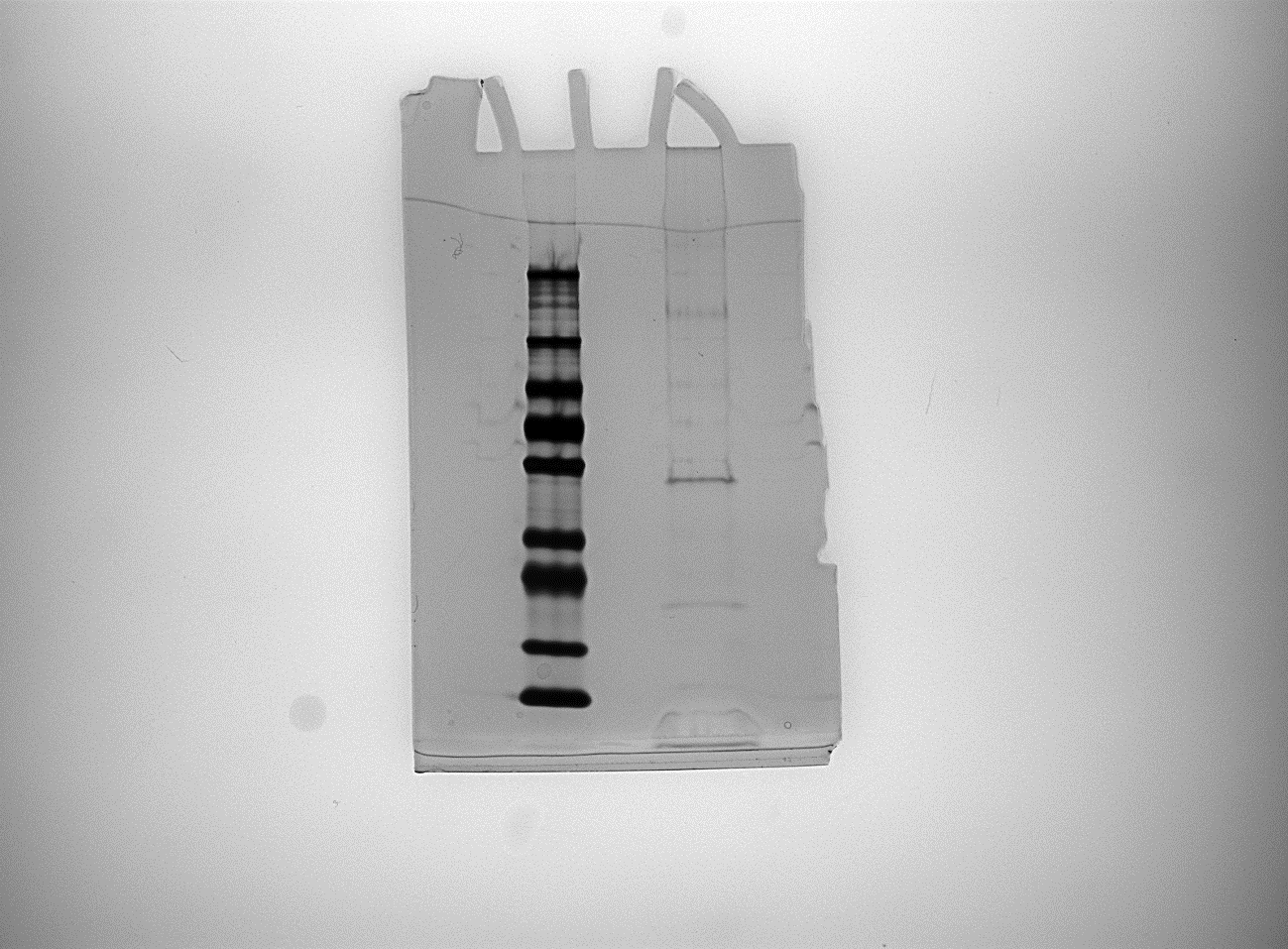


**A**

**B**

**Figure S7. Analysis of the purified and inactivated SARS-CoV-2.** Original figures for the SDS-PAGE [A] and Western blot strips [B], used to create Figure 1 presented on the main text.
